# Systematic evaluation of phage cocktail-ciprofloxacin combination therapy against multidrug-resistant *Salmonella* Typhimurium induced gut dysbiosis

**DOI:** 10.1128/spectrum.02640-25

**Published:** 2026-03-23

**Authors:** Qian Chong, Mingxia Cheng, Qing Cao, Jiabing He, Min Xiao, Yongguang Li, Zhonglong Wang, Jiayu Wang, Kunzhong Zhang, Huitian Gou

**Affiliations:** 1 College of Veterinary Medicine, Gansu Agricultural University 74661, Lanzhou, Gansu, China; 2 Gansu Provincial Animal Disease Prevention and Control Center, LanZhou, Gansu, China; 3 Dingxi Vocational and Technical College, Dingxi, Gansu, China; Universidad Maimonides, Buenos Aires, Argentina

**Keywords:** *S*. Typhimurium, phage cocktail-CIP, intestinal inflammation, gut microbiota

## Abstract

Phage therapy, despite its promise as a novel therapeutic alternative for multidrug-resistant (MDR) bacterial infections, is hampered by clinical limitations such as narrow host range and therapeutic inconsistency. This research has engineered a phage-antibiotic synergistic therapy enabling precision eradication of drug-resistant *Salmonella* Typhimurium (*S*. Typhimurium). From slaughterhouse wastewater and fecal samples, three *Felixounavirus* phages (TSP_TW2, TSP_SW1, and TSP_SJ5) were isolated. Compared to the clinical phage repository, these phages demonstrated more superior broad-spectrum activity, successfully lysing over 85% of the tested wild-type isolates. The *in vitro* study, validated by laser confocal fluorescence imaging demonstrates that compared to solitary phage, phage cocktail delays the emergence of resistance to 24 h while also achieving effective biofilm eradication. In the murine intestinal infection model, the experimental group treated with phage cocktail in combination with ciprofloxacin (CIP) demonstrated significant therapeutic effects, reaching 90% survival rate, restoring the CD4^+^/CD8^+^ T cell balance, and decreasing the levels of pro-inflammatory factors, which were significantly superior to those of phage or CIP alone. In addition, the susceptibility of *S*. Typhimurium to β-lactams, quinolones, and observed four- to eightfold reduction in tetracycline minimal inhibitory concentration (MIC) demonstrated effective restoration of antibacterial activity after therapeutic intervention. 16S rRNA high-throughput sequencing showed that the index of intestinal flora diversity of mice in the treatment group was not significantly difference to healthy control group, while specifically promoting the growth of beneficial microbial populations such as *Lactobacillus* spp. The innovative phage-antibiotic combination therapy established in this study demonstrated dual therapeutic advantages: effective clearance of antimicrobial-resistant strains and maintenance of enteric microbiome homeostasis, thereby offering a clinically promising strategy for controlling infections caused by antimicrobial-resistant *S*. Typhimurium.

**IMPORTANCE:**

Foodborne *Salmonella* infections threaten global public health, as conventional antibiotics accelerate resistance and disrupt microbial balance. We pioneer a synergistic phage-ciprofloxacin cocktail strategy that overcomes multidrug-resistant *Salmonella* infection through three key advances: First, it delays resistance evolution while eradicating biofilm matrices; second, the therapy synergistically enhances antibiotic sensitivity to restore efficacy of critical drugs; and third, the combined approach maintains optimal gut microbiota balance during pathogen clearance. By using environmentally derived phages with minimized antibiotic dosing, this strategy achieves targeted removal of resistant pathogens—including invasive biofilms—without collateral damage to commensal flora. Crucially, it prevents systemic inflammation and preserves intestinal barrier function. This ecologically sustainable paradigm provides a dual-defense mechanism against infections and microbiome dysbiosis, positioning phage-antibiotic synergy as a transformative tool for containing foodborne disease threats.

## INTRODUCTION

*Salmonella* continues to be a significant public health threat as a major foodborne pathogen, and data reveal that *Salmonella*-induced foodborne illnesses account for approximately 131 million cases annually (1), with confirmed diagnoses reaching several million and at least 370,000 fatalities globally (2). Annually, 91,000 cases are confirmed across EU Member States (3). In China, 1,134 *Salmonella* outbreaks (involving 89,050 cases, 1991–2022) revealed that foodborne transmission was the primary route of infection (72.1%), followed by waterborne spread (28.5%) and healthcare-facility infections (6.3%). Serotyping studies identified 84 serotypes across 13 serogroups, with *S*. Enteritidis, *S*. Typhi, and *S*. Typhimurium being the three most prevalent epidemic serovars (4). These pathogens are predominantly transmitted through contaminated water, milk, raw vegetables, seafood, and eggs (5, 6). Epidemiological surveillance data indicate a marked increase in the epidemiological dominance of *S*. Enteritidis in recent years, while *S*. Typhimurium continues to pose substantial outbreak risks. More alarmingly, the dissemination of MDR strains has progressively intensified containment challenges. These strains can form biofilms on food processing surfaces and medical devices, reducing the penetration efficiency of disinfectants, leading to recurrent infections and amplifying the risks associated with foodborne transmission (7).

The prevalence of *Salmonella* MDR strains poses a serious challenge to clinical treatment and urgently requires the development of novel antimicrobial agents. Research has revealed widespread resistance to critical-priority antibiotics including β-lactams (73.76%), quinolones (49.51%), and aminoglycosides (71.05%), with healthcare-associated isolates exhibiting the most robust resistant phenotypes. It is noteworthy that *S*. Typhimurium exhibits particularly high resistance to β-lactams (58.82% after excluding ampicillin), with persistently elevated resistance rates maintained over time (4). Coexisting with the problem of drug resistance is the microecological crisis caused by antibiotic misuse. Chronic and extensive misuse of antimicrobial agents can fundamentally reshape gut microbiota homeostasis, inducing persistent microbial dysbiosis and functional impairment. This process is characteristically accompanied by a marked loss of microbial diversity and compromised colonization resistance, while additionally constituting a potential reservoir for antimicrobial resistance development (8, 9). Furthermore, through intricate host-microbiota interactions, such perturbations can profoundly disrupt core physiological functions including digestive metabolism and immune regulation, ultimately exacerbating disease susceptibility (10–12). Consequently, developing novel strategies capable of effectively eliminating drug-resistant *Salmonella* while preserving intestinal microbiota homeostasis has emerged as a critical imperative.

The escalating clinical challenges posed by emerging resistant strains have positioned phage therapy as a pivotal frontier in novel antimicrobial development, owing to its exceptional host-specific recognition and precision lytic mechanisms. Currently, phage cocktail therapy has emerged as a promising approach for combating drug-resistant bacterial infections, targeting a wider range of *Salmonella* of different serotypes and providing a broader host range (13). Preclinical investigations utilizing standardized murine models demonstrate that topical phage therapy achieves complete clearance of gut bacterium acnes-induced acne-like lesions (14), while systemic administration effectively treats *Pseudomonas aeruginosa* pneumonia and sepsis (15) and controls hypervirulent *Klebsiella* pneumoniae bacteremia (16, 17). Furthermore, phage therapy has shown considerable promise in complex clinical scenarios, especially for MDR infections in burn wounds (18, 19), *Staphylococcus aureus* induced mastitis in murine models (20). effectively confirming its clinical applicability across diverse complex clinical scenarios. In particular, the phage-antibiotic synergy (PAS) approach has emerged as an innovative strategy against MDR pathogens, demonstrating unique clinical potential. Compared with conventional monotherapy, PAS not only enhances antimicrobial efficacy but also significantly delays the emergence of drug resistance by synergizing two different bactericidal mechanisms (21, 22).

Building upon these discoveries, we evaluated the efficacy of a Broad-Spectrum *Salmonella* Phage-Antibiotic Cocktail. Through systematic analysis of phage host ranges against *Salmonella* isolates, we successfully identified three broad-spectrum phages (TSP_TW2, TSP_SW1, and TSP_SJ5) with lytic activity, which were further characterized by transmission electron microscopy (TEM) and whole-genome sequencing. Subsequent screening of eight antibiotic classes identified CIP as the optimal synergistic partner for phage co-administration. This dual strategy overcomes the bottleneck of narrow phage host range and delays resistance development via precise antibiotic synergy, with no observable perturbations to murine gut microbial composition during prolonged intervention, thereby achieving concurrent infection control and microbiome homeostasis maintenance.

## MATERIALS AND METHODS

### Bacterial strains and culture conditions

Through systematic sampling, 73 *Salmonella* strains were isolated from environmental effluents of livestock and poultry slaughterhouses and farms covering the most common serotypes from clinical samples of livestock and poultry between 2018 and 2024, including *S*. Typhimurium, *S*. Pullorum, *S*. Choleraesuis, *S*. Typhi, *S*. Enteritidis, *S*. Derby, *S*. Rissen, *S*. Paratyphi C, for phage host range determination and one strain of *E. coli* was provided by the Veterinary Public Health Laboratory, College of Veterinary Medicine, Gansu Agricultural University. All *Salmonella* strains were cryopreserved in 50% glycerol at −80°C and then inoculated in Luria-Bertani (LB) broth medium prior to experiments. The cultures were grown to mid-logarithmic phase (OD_600_ = 0.6) at 37°C with shaking at 200 rpm for experimental use.

### Phage isolation, purification, and host range determination

Environmental samples from livestock/poultry slaughterhouses and farms (Table S1) were collected across Gansu Province. After pre-cooling at 4°C, samples were centrifuged (8,000 rpm, 20 min) and filtered through sterile 0.22 μm membranes. The phage enrichment system was established by mixing host bacterial suspension, sample filtrate, and LB liquid medium at a 1∶2∶4 ratio, followed by 8 h incubation (37°C, 150 rpm). Phages were isolated using the double-layer agar method, where 3 μL crude lysate was spotted onto plates containing the host bacterial strain (top layer: 0.5% LB semi-solid agar with host bacteria at OD_600_=0.6; bottom layer: 1.5% LB solid agar), followed by overnight incubation at 37°C. Plaques with distinct edges and translucent centers were selected and purified through successive 10-fold dilutions, with dilution ratios adjusted based on plaque density.

One hundred microliters of aliquot of the diluted sample was thoroughly mixed with equal volume of host bacterial suspension, followed by 10 min of adsorption at 37°C before top agar overlay. After incubation, individual plaques were picked and submerged in 500 μL SM buffer at 4°C to release phage particles. Repeat the gradient dilution with bilayer plate purification step 5 to 6 times to finally obtain purified phage. Plaque assay was employed for initial phage host range screening against the collection of 73 *Salmonella* wild strains. Purified phages were adjusted to 10^9^ PFU/mL in SM buffer, and 3 µL lysate was spotted onto upper agar surfaces containing *Salmonella* host bacteria. Bacteriolytic activity was evaluated according to a 0 to 4 scoring system (0: no lysis; 4: complete clearance), with the standardized data subsequently visualized as a heatmap.

### Transmission electron microscopy

Twenty microliters of aliquot of purified phage suspension was deposited onto 300-mesh carbon-coated copper grids (SolelyBio, China) and allowed to adsorb for 10 min at room temperature. Subsequently, 20 μL uranyl acetate stain was applied to the grids for 5 min before removing excess stain with filter paper and completing final drying under incandescent lighting. Phage morphology was analyzed using a JEM-1400 TEM (JEOL) operated at 80 kV, with images captured for morphological analysis.

### Whole genome sequencing and analysis

One hundred microliters of phage suspension was mixed with equal volume of host bacterial culture and incubated (37°C, 15 min), followed by growth in 100 mL LB with orbital shaking (37°C, 220 rpm, 8 h). Supernatant collected via centrifugation (8,000 rpm, 15 min, 4°C) and sterile-filtered (0.22 μm). The filtrate was treated with DNase I and RNase I (final concentration 1 μg/mL) at 37°C for 50 min, followed by addition of NaCl (final concentration 1 mol/L) and subsequent incubation on ice for 1 h. Repeated centrifugation was performed (4°C, 8,000 rpm, 10 min). Supernatant precipitated with 10% (wt/vol) PEG 8000 and then centrifuged (4°C, 10,000 rpm, 15 min) to collect the precipitate. The precipitate was resuspended in 1 mL SM buffer (0 mM Tris-HCl, 100 mM NaCl, 8 mM MgSO_4_, pH 7.5), dissociated at room temperature for 12–14 h, and then extracted with 1 mL of chloroform (5,000 rpm, 15 min 4°C). The hydrophilic phase containing phage particles was carefully collected after phase separation. To improve phage purity, chloroform extraction may be repeated 1–2 times. Phage suspension was filtered through 0.22 μm membrane to obtain concentrate. Phage genomic DNA was extracted from the concentrated stock using the FastPure Viral DNA/RNA Mini Kit (Vazyme Biotech, Nanjing), followed by whole-genome sequencing analysis on the PacBio Sequel II platform (Frasergen Bioinformatics, Wuhan, China). Using BWA (version 0.7.12) comparison software, clean data were compared to the reference genome sequence, the sequences on the comparison were extracted, and the assembly was completed using the software pb assembly microbial (SMRT Link 10.1.0). Open reading frames (ORFs) were predicted using the fast annotation feature in Pharokka (https://github.com/gbouras13/pharokka) (23). Multiple sequence alignment was performed using MUSCLE v3.8, followed by phylogenetic tree construction with the neighbor-joining method based on phage major capsid proteins in MEGA11. The final phylogenetic tree was visualized using iTOL v6.7.6 (https://itol.embl.de/).

### Phage cocktail preparation and *in vitro* bactericidal test

The phage cocktail was prepared by mixing equal volumes of TSP_TW2, TSP_SW1, and TSP_SJ5 suspensions (adjusted to 1 × 10^9^ PFU/mL). To assess the *in vitro* lytic capacity of the phage cocktail, 180 μL *Salmonella* suspension (10^7^ CFU/mL) representing eight serotypes was combined with 20 μL phage cocktail (10^9^ PFU/mL) in 96-well plates, achieving a multiplicity of infection (MOI) 10. The 96-well plate was incubated at 37°C with shaking at 160 rpm. Growth inhibition was monitored by OD_600_ (SpectraMax i3x) at 0–24 h intervals (24).

### Confocal laser scanning microscopy

To evaluate the inhibitory capability of phage cocktails against *Salmonella* biofilms, analysis was performed by CLSM combined with live/dead cell staining. After inoculating log-phase *S*. Typhimurium in 20 mm dishes for 24 h biofilm development, samples underwent 24 h treatment with TSP_SW1 phage or phage cocktail (1 × 10^9^ PFU/mL). Aspirate and discard the planktonic bacteria and clean with DPBS, after which biofilms were stained with SYTO9 (10 µM) and propidium iodide (30 µM) at 37°C for 20 min in light-avoidance conditions (25). Post-staining PBS washes preceded laser confocal microscopy for fluorescence image acquisition and quantitative analysis.

### Antimicrobial selection

We assessed antimicrobial susceptibility using the Kirby-Bauer disk diffusion assay (26). A panel of eight antibiotic classes was selected to evaluate changes in *Salmonella* susceptibility profiles before and after phage cocktail combination. The specific steps were as follows: fecal samples from phage-treated mice were homogenized in PBS, and individual *Salmonella* colonies were purified and cultured in LB broth to mid-log phase (OD_600_= 0.6). One hundred microliters of bacterial culture was spread evenly on LB agar plates. After brief drying, antibiotic sensitivity pieces from eight antimicrobial drugs were placed on the agar surface, followed by incubation at 37°C for 16 h for measurement of inhibition zone diameters. Sentence classification (S), intermediate (I), or resistant (R) was performed for each antibiotic against the bacterial strains according to the Clinical and Laboratory Standards Institute (CLSI) M100 guidelines, thereby enabling a systematic evaluation of the impact of phage intervention on the antibiotic resistance profile of *Salmonella*.

### Mouse experiments

Male-specific pathogen-free Kunming mice (age 6–8 weeks) were provided by Chinese Academy of Agricultural Sciences-affiliated Lanzhou Veterinary Research Institute’s animal facility. All procedures were approved by the Animal Welfare and Research Ethics Committee of Gansu Agricultural University (approval no. GSAU-Eth-VMC-2025-039) and conducted in compliance with Chinese National Standards for Laboratory Animals (27). After 3 days of acclimation to the experimental environment, mice were challenged with *Salmonella* and treated with different phage groups. Fresh fecal samples were then collected from all groups every 12 h. Animals were randomly divided into five groups (*n* = 10 per group): (i) negative control receiving 300 μL PBS by oral gavage, (ii) infection control administered 300 μL *S*. Typhimurium CMCC 50115 suspension (10^8^ CFU/mL), (iii) phage therapy group receiving 300 μL bacterial suspension followed after 1 h by 300 μL phage cocktail (10^9^ PFU/mL), (iv) antibiotic therapy group administered 300 μL bacterial suspension followed after 1 h by 300 μL CIP, and (v) combination treatment group: Administer 300 μL of bacterial suspension, followed by 150 μL of phage mixture, and then administer 150 μL of CIP after 1 h. Mice were monitored at 24 h intervals for survival and body weight changes over 7-days observation period. After 24 h of phage treatment, three mice per group were randomly dissected. Under aseptic conditions, the liver, heart, thymus, kidneys, and intestines were collected for organ index determination. Pathologically significant intestinal segments, spleen, and liver were homogenized, and bacterial loads were quantified using the serial dilution plating method. Following fixation in 10% formalin, tissues were embedded, sectioned, and subjected to H&E staining for histopathological examination. To evaluate *in vivo* phage activity, anal swab samples were collected every 12 h post-treatment using sterile cotton swabs moistened with PBS. After centrifugation (12,000 rpm, 10 min), supernatants were 0.22 μm membrane-filtered, and recovered phage titers were quantified by the double-layer agar assay.

Peripheral immune cell subsets were analyzed by flow cytometry using 125 μL blood. Samples were divided into three groups: unstained negative controls, single-color compensation controls, and test samples stained with 5 μL each of PE-anti-CD3ε, PerCP-Cy5.5-anti-CD4, and FITC-anti-CD8 antibodies. After vortex mixing, samples were incubated for 15 min at room temperature in the dark prior to acquisition. The pellets were resuspended in 1 mL PBS and centrifuged at 3,000 rpm for 5 min before final supernatant removal. Cells were resuspended in 500 μL of flow cytometry buffer, filtered through a 300-mesh nylon membrane, and analyzed by flow cytometry. Samples were protected from light at 2–8°C and acquired within 24 h using a BD FACSCanto II instrument. CD3^+^CD4^+^ and CD3^+^CD8^+^ T cell populations were quantified using FACSDiva software. For simultaneous assessment of systemic immune response, serum was separated by centrifugation of peripheral blood at 3,000 rpm for 10 min, and the levels of inflammatory cytokines, such as IL-6, TNF-α, and IL-1β, were quantitatively detected according to the instructions of mouse ELISA Kit (YUANJU Bio).

### 16S rRNA gene sequencing and analysis

Cecal contents were collected post-dissection and five representative samples per group (five groups total) were immediately flash-frozen in liquid nitrogen for long-term storage at −80°C. For processing, 500 mg of thawed samples was homogenized with PBS. Pellet bacteria by centrifugation (8,000 rpm, 10 min), resuspend in 500 μL lysis buffer (500 mM NaCl, 50 mM Tris-HCl, 50 mM EDTA, 4% SDS), incubate 50℃ for 15 min. Add 100 μL lysozyme (25 mg/mL) and incubate 37℃ for 2 h. Total microbial DNA was extracted using the CretMag Power Soil DNA Kit (Cretage Biotechnology, China) strictly following the manufacturer’s protocols. Polymerase Chain Reaction (PCR) amplification targeting the V3-V4 region of the bacterial 16S rRNA gene was performed using forward primer 341F (5′-ACTCCTACGGGGAGGCAGCA-3') and reverse primer 806R (5′-GGACTACHVGGGGTWTCTAAT-3′) with 30 amplification cycles. The PCR amplicons were quantified using the QuantiT PicoGreen dsDNA Assay Kit (Invitrogen, Carlsbad, CA, USA) and subsequently sequenced on the Illumina NovaSeq PE250 platform (2,250 bp paired-end reads). Data processing was performed using QIIME (version 1.8.0; http://qiime.org) and VSEARCH (version 2.7.1; https://github.com/torognes/vsearch) for quality filtering, operational taxonomic unit (OTU) clustering, and taxonomic annotation. Multivariate statistical analyses, principal component analysis (PCA), were conducted using the R programming environment.

### Statistical Analysis

All statistical analyses were performed using GraphPad Prism version 8.2.1. Experiments were conducted in triplicate, with statistical significance determined by one-way analysis of variance (ANOVA) followed by Tukey’s multiple comparisons test. Data were analyzed by the Gehan-Breslow-Wilcoxon method in the Kaplan-Meier survival analysis. Data are presented as mean ± standard deviation (SD), with statistical significance defined as *P* < 0.05 (*P < 0.05; **P < 0.01; ***P < 0.001; ns, not significant *P* > 0.05).

## RESULTS

### Isolation and characterization of phages TSP_SJ5, TSP_SW1, and TSP_TW2

**Fig 1:**
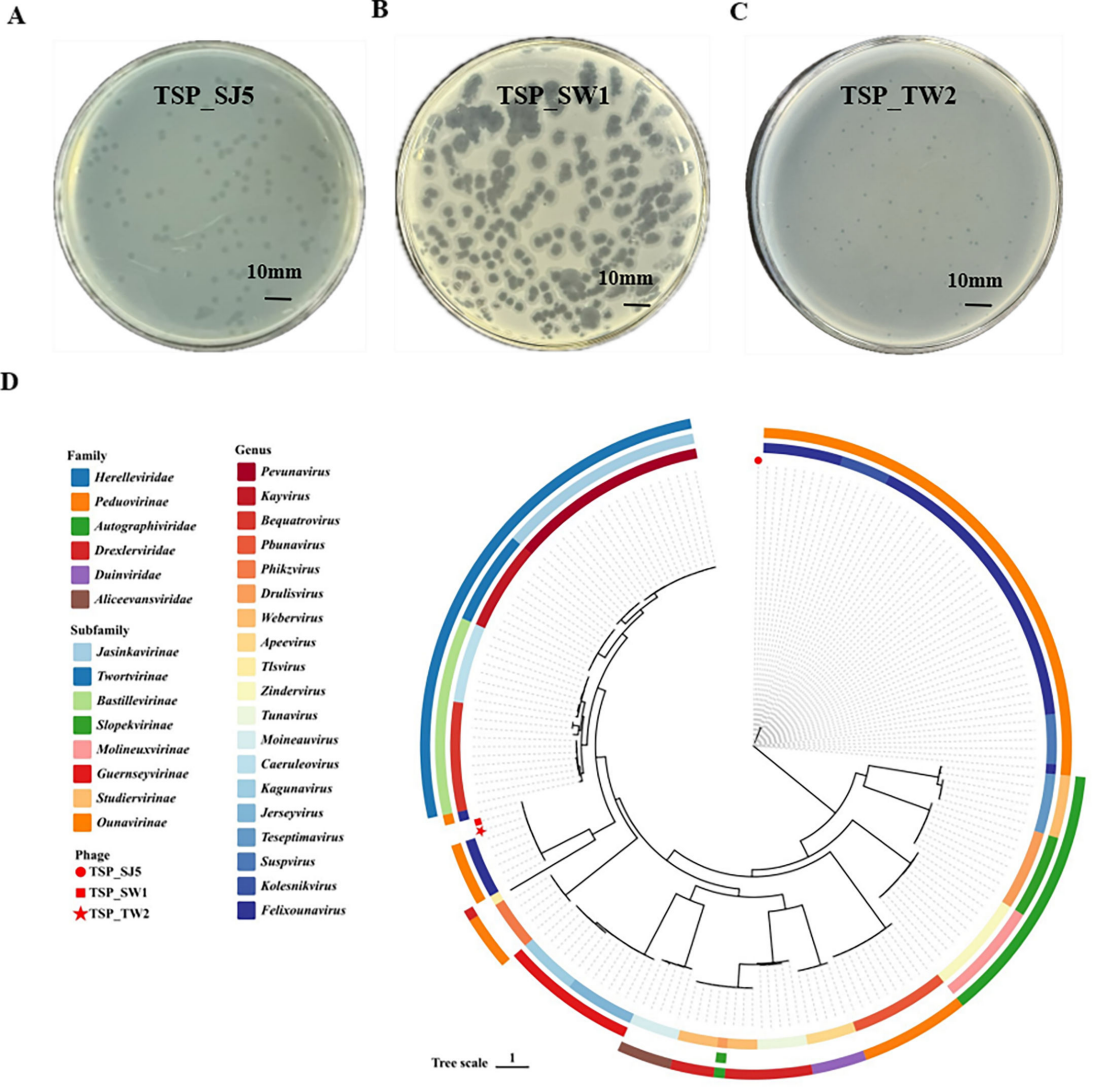
Characteristics of phages TSP_SJ5, TSP_SW1, and TSP_TW2. (**A**) Plaque morphology of TSP_SJ5 on LB plates. (**B**) Plaque morphology of TSP_SW1 on LB plates. (**C**) Plaque morphology of TSP_TW2 on LB plates. (**D**) Phylogenetic analysis based on the major capsid protein constructed using ViPTree, with taxonomic families/subfamilies indicated in the outer ring and genera highlighted in the inner ring. The phages TSP_SJ5, TSP_SW1, and TSP_TW2 are marked by red circles, red squares, and red pentagrams, respectively.

*S*. Typhimurium CMCC 50115 was, therefore, selected as the target in this study. All three phages formed clear and transparent plaques. TSP_SJ5 plaques were 1–1.5 mm in diameter, and TSP_TW2 plaques were 0.8–1 mm in diameter. Among them, TSP_SW1 produced characteristic central plaques (1–2 mm in diameter) surrounded by halo zones (3–6 mm in width), consistent with the lysis pattern of virulent *Salmonella* phages (Fig. 1A through C) (Table S2). Morphological analysis revealed that all phages exhibited typical Caudovirales morphology, each consisting of an icosahedral head and a contractile tail. The head diameter measured 88 ± 2 nm, while the tail length was 170 ± 2 nm (Fig. S1). Biological characterization revealed that all three phages retained high activity at 60°C and across pH 2–13 (Fig. S2A and B). One-step growth curve analysis demonstrated that TSP_SJ5, TSP_SW1, and TSP_TW2 exhibited stable replication characteristics, featuring latent periods all under 18 min. The burst sizes (representing the number of phage particles released per infected bacterial cell) were approximately 237, 165, and 247 PFU/cell for TSP_SJ5, TSP_SW1, and TSP_TW2, respectively, titers reaching above 1 × 10^10^ PFU/mL (Fig. S2C). The MOI was 0.1 for TSP_SJ5 and TSP_SW1, and 1 for TSP_TW2 (Fig. S2D). Genome sequencing revealed that TSP_SJ5, TSP_SW1, and TSP_TW2 have total genome lengths of 86,053 bp, 86,647 bp, and 85,951 bp, with GC contents of 38.81%, 39%, and 38.80%, respectively (Fig. S3). Genomic collinearity analysis revealed that TSP_SW1 and TSP_ TW2 share a highly conserved structure, displaying a single collinear block across the entire genome, with nucleotide homology exceeding 95% and only minor variations in non-coding regions. In contrast, TSP_SJ5 exhibits significant structural rearrangements, forming four local collinear blocks, with structural changes in its tail gene cluster (45–52 kb). Despite these differences, the core structural genes (such as those for DNA packaging and capsid/tail proteins) are strictly conserved across all three phages, demonstrating the evolutionary stability of functional modules (Fig. S4). Genome sequencing data were deposited in the NCBI database with accession numbers (NCBI: PV550667, PV550668, and PV550669). The DeePhage prediction classified all phages as strictly lytic in lifestyle. Genome annotation confirms that TSP_SJ5, SW1, and TW2 all encode critical protein systems: lysis machinery proteins, structural proteins for virion assembly, DNA processing proteins, host regulatory factors, and other functional proteins, as detailed in Tabels S3 through S5. Moreover, systematic database searches were conducted using CARD (Comprehensive Antibiotic Resistance Database) and VFDB (Virulence Factor Database), no lysogenic genes, antibiotic resistance genes, or virulence factor genes were identified in the phage genomes. Phylogenetic analysis based on the major capsid protein indicated that TSP_SJ5, TSP_SW1, and TSP_TW2 are classified under Caudoviricetes class and *Felixounavirus* genus according to the latest International Committee on Taxonomy of Viruses (ICTV) classification (Fig. 1D).

### Host range determination of the phages

**Fig 2:**
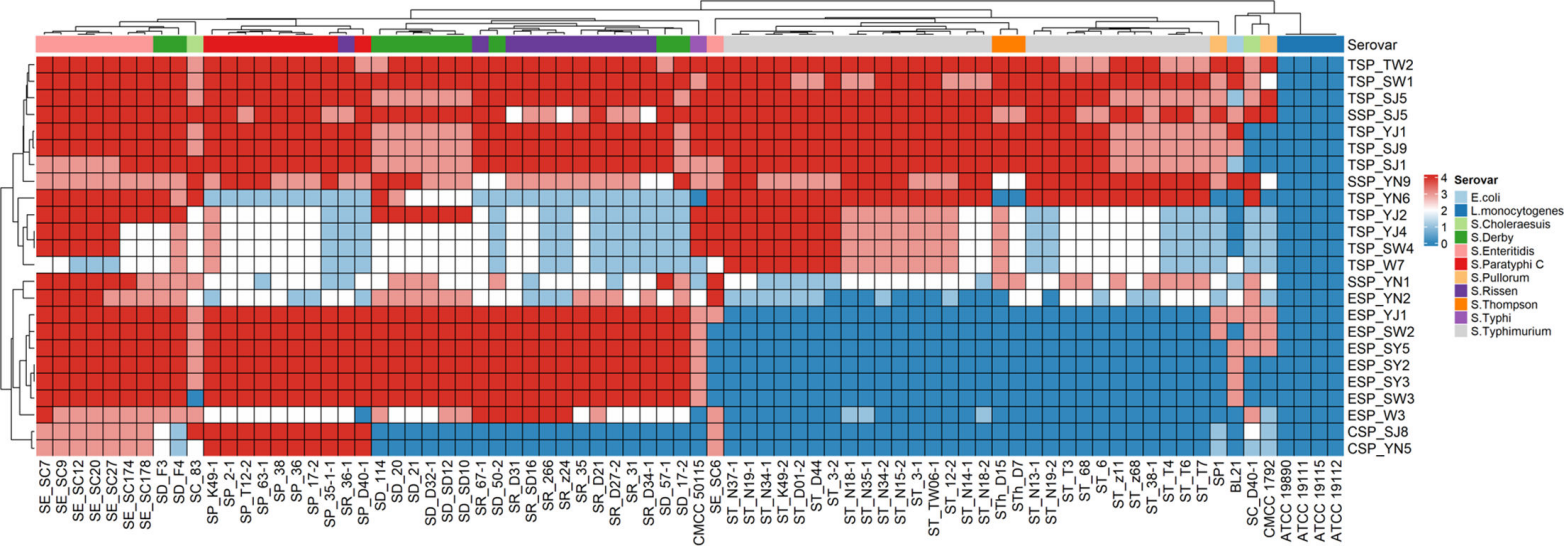
Cluster analysis of isolated phages’ host range and lytic activity profiles. Lytic activity was evaluated using a semiquantitative grading system: “0” (no lytic activity, dark blue) indicating no visible clearance zone; “1” (weak activity, light blue) showing only opaque turbid zones; “2” (partial lysis, white) with distinct yet turbid halos; “3” (significant lysis, light red) displaying nearly clear zones with slight background turbidity; and “4” (complete lysis, dark red) exhibiting well-defined, fully transparent plaque.

Screening identified three *Salmonella* phages (TSP_TW2, TSP_SJ5, and TSP_SW1) demonstrating >75% lytic efficiency against MDR strains, among which TSP_TW2 exhibited the broadest host range. This phage lysed eight *Salmonella* serovars—including *S*. Pullorum, *S*. Enteritidis, and *S*. Typhimurium—along with select *E. coli* strains at 85% efficiency (Fig. 2), while TSP_SJ5 and TSP_SW1 achieved 77% and 83% efficiency, respectively. Given their potent lytic activity and expansive host spectra, particularly the cross-genus activity of TSP_TW2, these phages represent promising candidates for broad-spectrum phage therapy development. Consequently, they were selected for further characterization.

### *In vitro* killing curves of *Salmonella* phage cocktails

**Fig 3:**
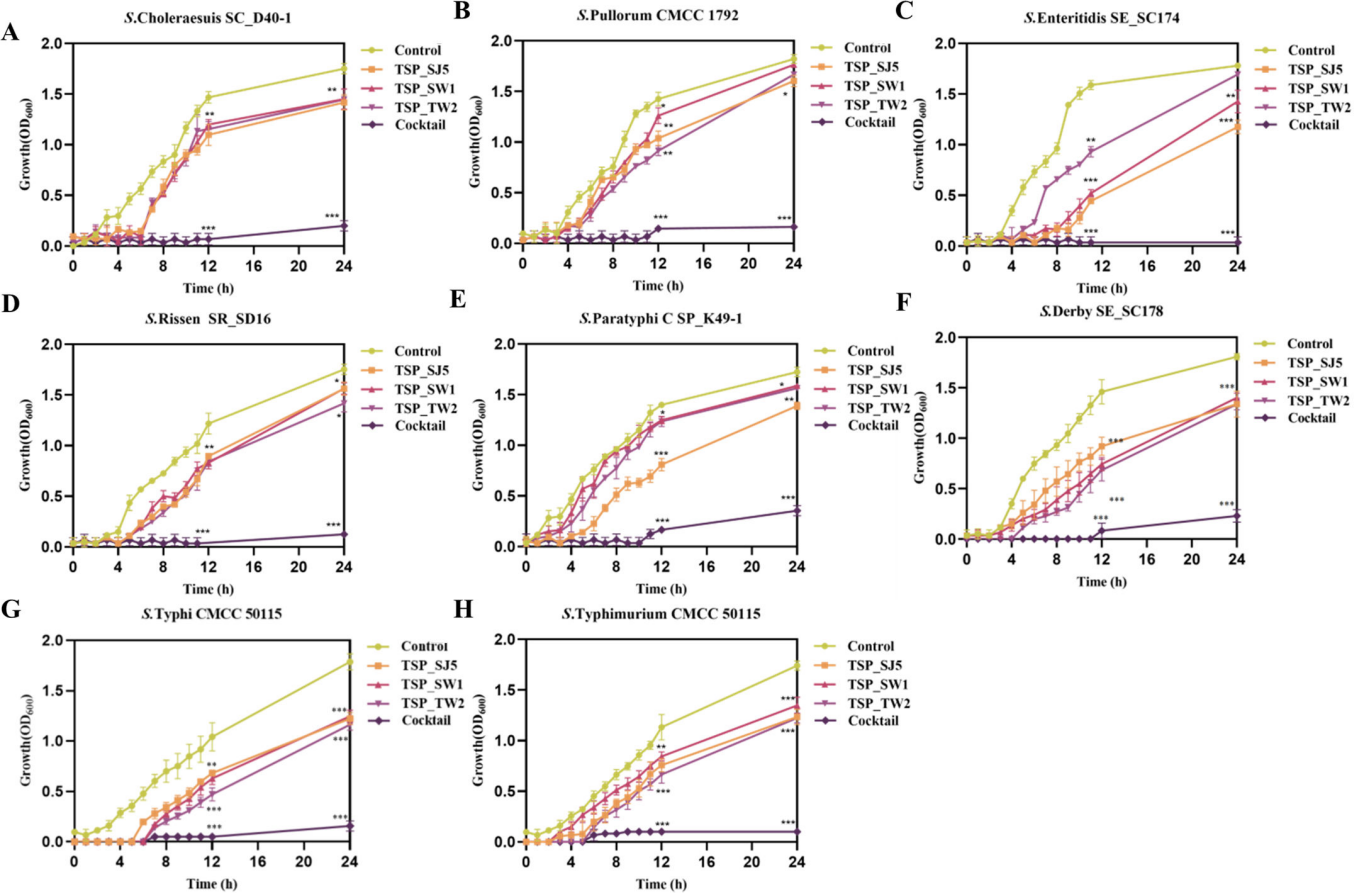
*In vitro* killing curves of single phages versus phage cocktail over 24 h. (**A**) *S. Choleraesuis* SC_D40-1. (**B**) *S. Pullorum* CMCC 1792. (**C**) *S. Enteritidis* SE_SC174. (**D**) *S. Rissen* SR_SD16. (**E**) *S. Paratyphi* C SP_K49-1. (**F**) *S. Derby* SE_SC178. (**G**) *S. Typhi* ST_TW06-1. (**H**) *S.* Typhimurium CMCC 50115. All experiments were performed in triplicate, with error bars representing standard deviation. Data were analyzed by one-way ANOVA followed by Tukey's post hoc test (ns, not significant; *P < 0.05; **P < 0.01; ***P < 0.001).

To systematically analyze the *in vitro* antimicrobial effects of phage combinations, eight MDR *Salmonella* strains (representing common serovars) were selected for growth curve monitoring via optical density (OD_600_), with comparative analysis of killing efficacy between single phage and phage cocktails over a 24 h period. This timeframe was chosen based on *Salmonella*’s peak susceptibility to phage predation during the logarithmic growth phase. The phage cocktail demonstrated superior bactericidal efficiency compared to all individual component phages across the eight tested strains. The results indicated that most strains developed resistance to individual phages within 4–6 h. In contrast, the phage cocktail composed of TSP_SJ5, TSP_SW1, and TSP_TW2 achieved significantly stronger and more sustained lytic activity (12–24 h) through synergistic effects. Statistical analysis confirmed that the antibacterial efficacy of the cocktail treatment group showed highly significant differences compared to the control group at key time points, thereby effectively delaying the emergence of bacterial resistance (Fig. 3).

### CLSM reveals the anti-biofilm activity of phage cocktails

**Fig 4:**
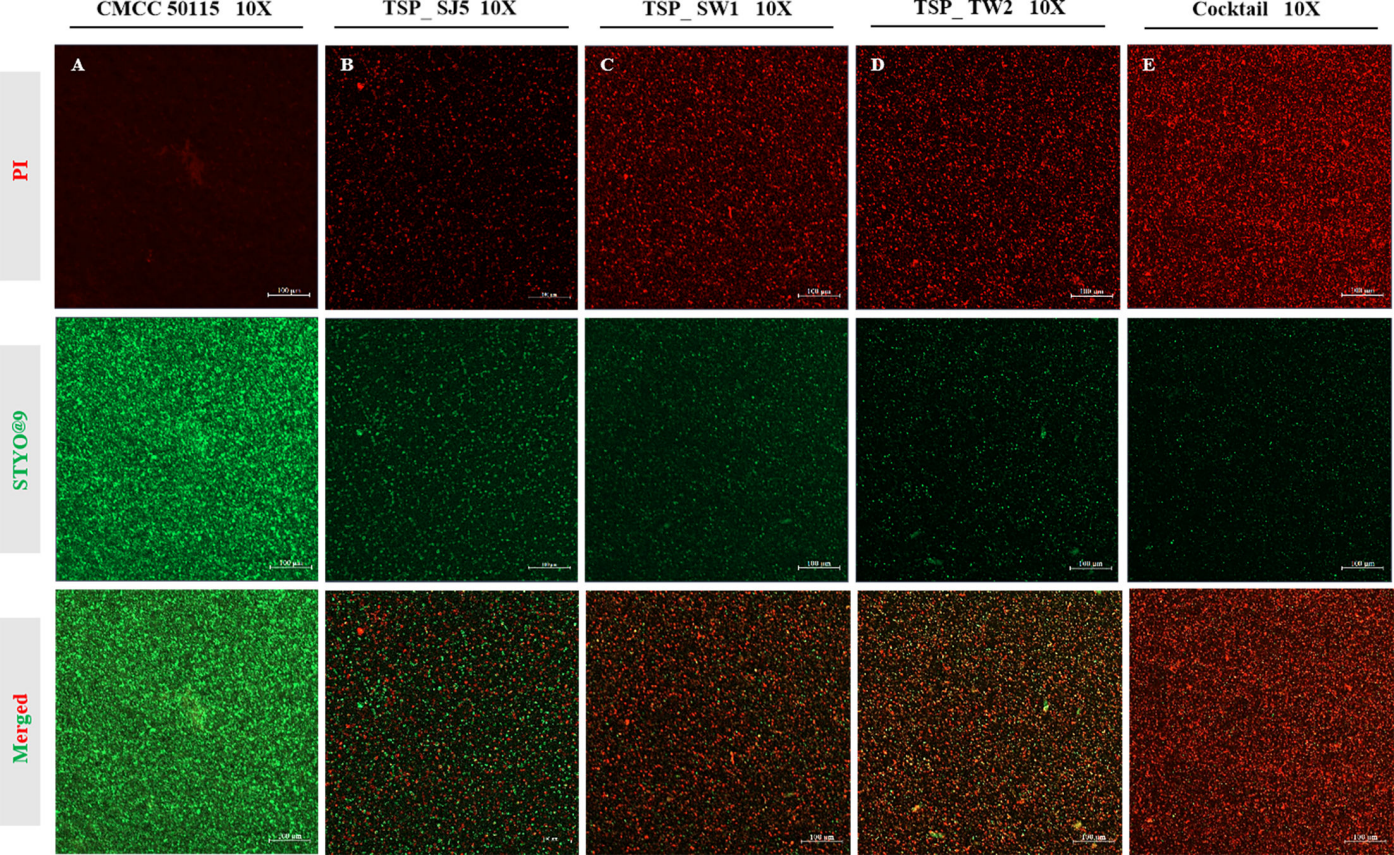
The clearance efficiency of phage on biofilms. Dual-stained (green = viable cells, SYTO 9; red = dead cells, propidium iodide) 24 h mature biofilm of CMCC 50115 analyzed by CLSM: (**A**) Untreated control. (**B**) TSP_SJ5-treated. (**C**) TSP_SW1-treated. (**D**) TSP_TW2-treated. (**E**) Phage cocktail-treated group.

Through CLSM imaging analysis (Fig. 4), propidium iodide (PI)-labeled dead bacterial cells exhibited red fluorescence, while SYTO 9-labeled live bacterial cells displayed green fluorescence. In the untreated *S*. Typhimurium biofilm control group, persistent dominant green fluorescence signal (live bacterial density >96%) indicated that the bacterial cells were in a complete physiological state (Fig. 4A). After phage cocktail therapy (Fig. 4E), biofilm exhibited a significant dominance of red fluorescence throughout the entire area, with a significantly higher red fluorescence intensity compared to the single phage TSP_SJ5, TSP_SW1, and TSP_TW2 treatment groups (Fig. 4B through D). This cocktail therapy demonstrated superior matrix penetration ability compared to single phages, with its enhanced lytic activity significantly improving bacterial clearance within the biofilm.

### *In vivo* efficacy of phage cocktail therapy in infected mice

Microbroth dilution assays (Fig. S5) demonstrated stable phage titers when CIP was combined with phages, with the MIC decreasing significantly from 4 μg/mL to 1 μg/mL. The tetracycline combination group showed an MIC reduction from 8 μg/mL to 4 μg/mL, indicating that (i) CIP-phage cocktail coadministration produced significant synergistic antibacterial effects (*P* < 0.05); (ii) the final inhibitory concentration could be reduced to 1 μg/mL. Based on superior synergy and lower MIC values, CIP was identified as the preferred antimicrobial for combination therapy with phage cocktails.

**Fig 5:**
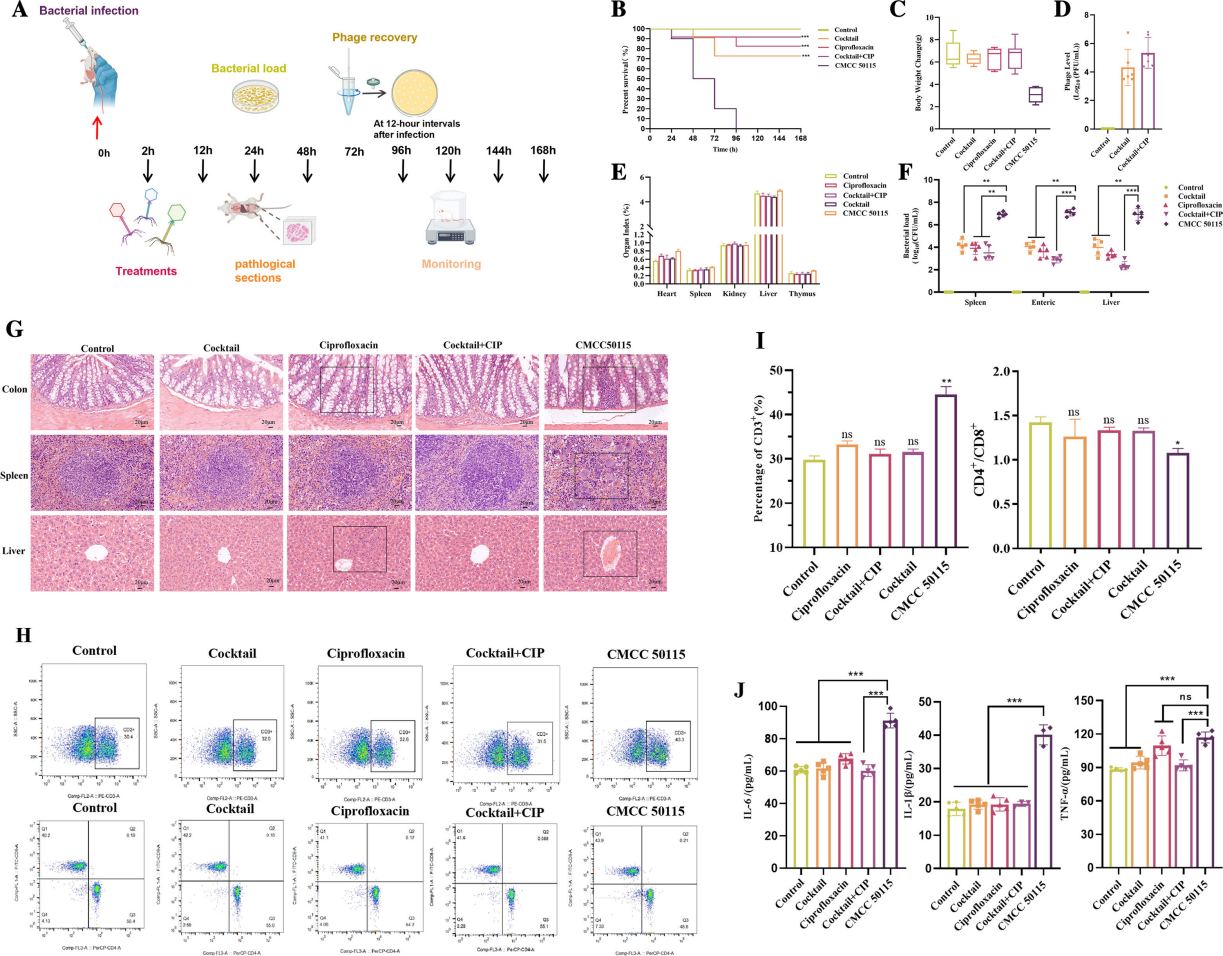
Therapeutic efficacy of phage cocktail against *S*. Typhimurium CMCC 50115 infection in a murine intestinal model. (**A**) Schematic of experimental protocol showing bacterial challenge at 0 h following 3-day acclimatization period. (**B**) Survival curves over 7-day observation period (*n* = 10 mice per group). (**C**) Body weight change monitored daily for 7 days post-infection (Δweight = final weight - initial weight). (**D**) Fecal phage recovery titers in mice receiving phage cocktail alone or combined with antibiotics. (**E**) Organ indices of heart, spleen, kidney, liver, and thymus across treatment groups. (**F**) Bacterial loads (CFU/g) in spleen, intestinal and liver tissues following phage-based interventions. (**G**) Histopathological examination of colon, spleen, and liver sections stained with H＆E (400× magnification; analyzed using SIideViewer software). (**H**) Flow cytometric analysis results of peripheral blood lymphocyte subsets (**I**) CD3^+^ T cell percentages and CD4^+^/CD8^+^ ratios. (**J**) Serum concentrations of inflammatory cytokines (IL-1β, IL-6, TNF-α) compared to uninfected controls. All experiments performed in triplicate; error bars represent standard deviation. Statistical analysis by one-way ANOVA with Tukey's post-hoc test (ns: not significant; *P < 0.05; **P < 0.01; ***P < 0.001).

A mouse intestinal infection model was established to validate the therapeutic efficacy of phage cocktail administration alone or in combination with antibiotics *in vivo* (Fig. 5A). All mice in the untreated infected group died within 96 h (Fig. 5B). In contrast, the phage cocktail monotherapy group achieved 70% survival rate, while CIP alone yielded 80% survival. The phage cocktail-CIP combination therapy further elevated survival to 90%, demonstrating statistically significant superiority over individual treatments (*P* < 0.01). Body weight monitoring demonstrated that the phage-antibiotic combination therapy restored growth progression in *Salmonella* infected mice (Fig. 5C). To assess the viability and colonization capacity of orally administered phages in the intestinal tract, fecal phage recovery titers were measured in mice. The combination therapy group maintained a phage titer of 5.9,× 10^6^ PFU/mL at 12 h, followed by rapid decline during 12–24 h before stabilizing at 3.7 × 10^4^ PFU/mL, demonstrating effective oral delivery of viable phages to the gut with functional colonization maintenance (Fig. 5D). Pathological alterations in immune organs (spleen, thymus) and metabolic organs (liver, kidneys) post infection, organ index analysis (Fig. 5E) revealed that *Salmonella* infected mice exhibited significantly increased liver indices (*P* < 0.05) with nonsignificant enlargement of the spleen and thymus, whereas kidney indices showed no intergroup differences. To determine whether the phage-antibiotic combination could reduce bacterial loads in major tissues, quantitative analysis of histopathological specimens was performed (Fig. 5F). Results demonstrated that phage monotherapy significantly decreased bacterial counts in the intestine, liver, and kidneys compared with the infection control group (*P* < 0.01). The results showed that both the phage-alone group and the antibiotic-alone group exhibited significant therapeutic effects, with the average bacterial load decreasing by approximately 2–3 log10. In contrast, the cocktail-antibiotic combination group demonstrated a more superior pathogen clearance efficacy, as the average bacterial load decreased by more than 4 log10 in all examined tissues (*P* < 0.001). This ultimately validated the rapid and significant synergistic therapeutic effect of the combined regimen *in vivo*. Histopathological analysis by H&E staining was performed on the colon (primary infection site), spleen (immune-responsive organ), and liver (metabolic detoxification organ) to characterize tissue damage induced by CMCC 50115 infection and therapeutic outcomes (Fig. 5G). Inflammatory cell infiltration severity and tissue injury patterns were systematically compared across groups. Compared to controls, infected mice exhibited colonic epithelial architecture disruption, crypt deformation, disorganized goblet cell arrangement, and extensive inflammatory cell infiltration. In the phage cocktail treated group, the epithelial structure remained relatively intact with no significant pathological changes observed. The antibiotic treated group exhibited incomplete colonic epithelial structure, accompanied by extensive inflammatory cell infiltration and significant pathological changes. Mice treated with the phage-antibiotic combination exhibited restored colonic epithelial architecture with tightly arranged goblet cells and absence of inflammatory infiltration. Liver sections from infected controls displayed central vein congestion, protein exudation, and inflammatory cell infiltration. The hepatic cord in the phage-treated group was arranged in an orderly manner, with no evidence of inflammatory cell infiltration. The central vein in the antibiotic group showed slight protein exudation and was accompanied by extensive inflammatory cell infiltration. Combination-treated mice displayed orderly hepatic cord arrangements with intact lobular architecture and no discernible abnormalities. Infected controls exhibited marked splenic mononuclear macrophage proliferation, indistinct germinal center boundaries, and structural disorganization. The germinal centers in the bacteriophage-treated group exhibited well-defined boundaries and showed no significant pathological abnormalities. The germinal centers in the antibiotic group were disorganized and accompanied by mild inflammatory cell infiltration, which were normalized post combination therapy, showing well-demarcated germinal centers without inflammatory infiltration. These findings confirm that phage cocktail-antibiotic combination therapy alleviates intestinal inflammation, preserves gut barrier integrity, mitigates systemic organ damage, and reduces *S*. Typhimurium-induced intestinal pathology. Flow cytometric analysis (Fig. 5H) demonstrated that *S*. Typhimurium infection induced significant immune dysregulation, marked by elevated CD3^+^ T cell frequencies (*P* < 0.001) and reduced CD4^+^/CD8^+^ ratios (*P* < 0.05). Demonstrated that *S*. Typhimurium infection induced significant immune dysregulation, marked by elevated CD3^+^ T cell frequencies (*P* < 0.001) and reduced CD4^+^/CD8^+^ ratios (*P* < 0.05). Phage cocktail, antibiotic monotherapy, and combination treatment all restored CD3^+^ cell homeostasis and normalized CD4^+^/CD8^+^ balance to levels comparable with uninfected controls (Fig. 5I). ELISA revealed significantly elevated serum levels of TNF-α, IL-6, and IL-1β in the infection group compared to the control group (*P* < 0.01). The combination therapy group reduced cytokine levels to near those of the control group compared to the other treatment groups (Fig. 5J). These results indicate that the phage cocktail-antibiotic combination therapy effectively alleviated the *S*almone*lla* induced inflammatory response, demonstrating an optimal anti-inflammatory effect.

Existing studies indicate that antibiotics can modulate phage lytic activity through either synergistic or antagonistic pathways (21, 28). This research further investigates whether similar interaction patterns exist between the phage cocktail recovered from mouse feces post-treatment and conventional antibiotics. Using the disk diffusion method, we examined changes in the inhibition zone diameters of antibiotic-resistant *S*. Typhimurium CMCC 50115 strains (Table 1). Relative to the untreated control group, 50% of tested antimicrobials showed significantly larger inhibition zones (*P* < 0.05). Notably, the susceptibility profile shifted from resistant to sensitive for aminoglycosides (streptomycin), and from intermediate to sensitive for polymyxins (polymyxin B), β-lactams (cephalexin), and tetracyclines (tetracycline, minocycline). Additionally, cephalosporins (cephazolin, ceftriaxone) and quinolones (CIP, ofloxacin) demonstrated significantly increased inhibition zones, suggesting better therapeutic efficacy compared with single-antibiotic treatment groups.

**TABLE 1:** Antibiotic susceptibility test results of *S*. Typhimurium CMCC 50115

Type		Antibiotic	Resistant (R)	Intermediate (I)	Susceptible (S)	Inhibition zone diameter	Inhibition zone post-phage
Beta-lactams	Penicillins	Ampicillin	≤13	14–16	≥17	22	23
		Cefazolin	≤14	15–17	≥18	20	23
	Cephalosporins	Cephalexin	≤14	15–17	≥18	18	18
		Ceftriaxone	≤13	14–20	≥21	26	30
Aminoglycosides		Streptomycin	≤11	12–14	≥15	10	15
		Kanamycin	≤13	14–17	≥18	18	18
		Gentamicin	≤12	13–14	≥15	18	21
		Amikacin	≤14	15–16	≥17	20	20
Polymyxins		Polymyxin B	≤10	11–15	≥16	15	16
Macrolides		Erythromycin	≤12	13–14	≥15	0	0
Lincosamides		Lincomycin	12	13–20	≥21	0	0
Glycopeptides		Vancomycin	≤12	13–14	≥15	0	0
Quinolones		Ciprofloxacin	≤15	16–20	≥21	24	30
		Ofloxacin	≤14	15–19	≥20	23	26
Tetracyclines		Tetracycline	≤14	15–18	≥19	15	26
		Minocycline	≤14	15–18	≥19	14	17

### Effect of phage cocktail on gut microbial diversity

**Fig 6:**
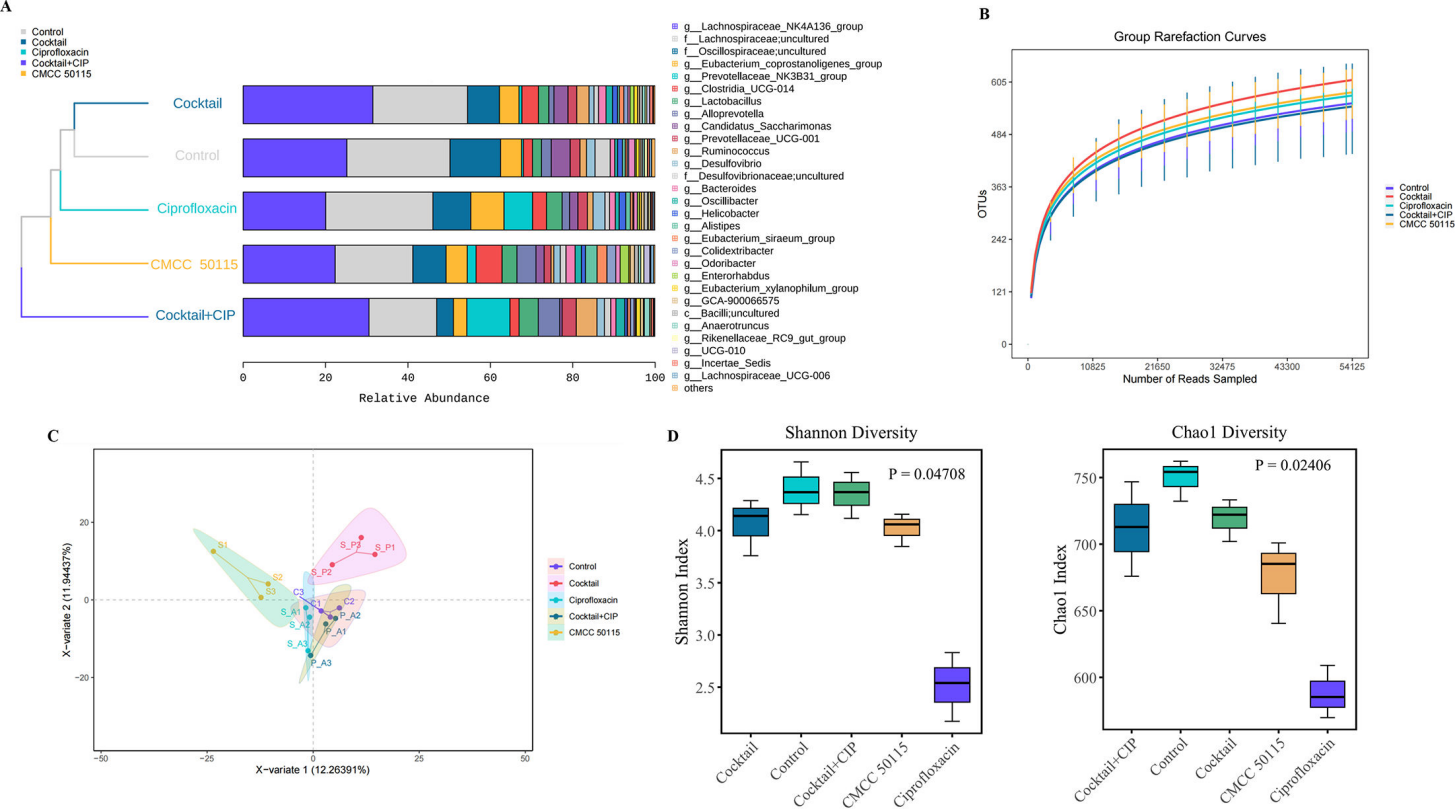
Effects of different treatments on murine cecal microbiota diversity and composition. (**A**) Genus-level Bray-Curti’s similarity clustering of gut bacterial communities (UPGMA clustering based on unweighted Unifrac distance matrix). (**B**) Cecal microbiota rarefaction curves generated by QIIME2. (**C**) Principal coordinate analysis (PCoA) of 15 metagenomes from five treatment groups at genus level, with explained variance percentages indicated on axes; points represent individual samples colored by treatment group. (**D**) α-Diversity analysis using Chao1 (richness estimator) and Shannon (diversity index) metrics for microbial community comparisons across groups. Error bars denote standard deviation (one-way ANOVA with Tukey's *post hoc* test; ns = not significant, *P < 0.05, **P < 0.01, ***P < 0.001).

To investigate the impact of phage therapy on gut microbiota homeostasis during targeted clearance of *S*. Typhimurium, this study systematically analyzed changes in intestinal microbial diversity across different treatment groups using 16S rRNA sequencing. First, the species accumulation curve reached a plateau at *n* = 6 (Fig. 6B), and the Chao1 indices (484–605) demonstrated that the sampling depth adequately captured gut microbial diversity. PCA analysis (Fig. 6C) revealed differences in gut microbiota composition among groups, with spatial distances reflecting community similarity. The infection group showed maximal separation from the control group, confirming that *S*. Typhimurium infection significantly altered gut microbiota structure. The phage cocktail-antibiotic combination group remained closely clustered with the control, demonstrating that this combined approach effectively inhibited the pathogen while better preserving original microbial community composition. Bray-Curti’s similarity clustering analysis (Fig. 6A) demonstrated that the mouse gut microbiota was predominantly composed of Firmicutes, Bacteroidetes, and Proteobacteria (Fig. S6). *Salmonella* infection significantly reduced the abundances of Firmicutes and Bacteroidetes (*P* < 0.05) while increasing Proteobacteria (*P* < 0.01). Phage cocktail treatment, particularly in combination therapy, effectively restored microbial balance, bringing Bacteroidetes abundance close to normal levels (*P* < 0.05). At the genus level, the phage-antibiotic treatment group exhibited marked reductions in potential pathogens (e.g., *Enterobacter*, *Desulfovibrio*, and *Helicobacter*) alongside increased abundances of probiotic genera *Lactobacillus* and *Bacteroide*s (24.5% and 29.8%, respectively). Furthermore, no significant differences were observed in Chao1 and Shannon indices among the control group, phage cocktail group, and phage-antibiotic combination group (*P* ＞ 0.05), indicating that phage application did not disrupt overall microbiota composition (Fig. 6D). In contrast, the antibiotic-treated group exhibited significantly lower Chao1 and Shannon indices compared to all other groups (*P* < 0.001), demonstrating that broad-spectrum antibiotics markedly reduced microbial community richness and diversity. This non-selective bactericidal effect may pose long-term risks of dysbiosis, including potential opportunistic pathogen proliferation or compromised metabolic functions.

## DISCUSSION

The rise of antimicrobial-resistant (AMR) *Salmonella* variants is creating substantial risks to both food security and population health on a global scale (29–31), significantly compromising the efficacy of existing antibiotic therapies while impeding the development of novel antimicrobial agents. Phage cocktails are widely regarded as potentially therapeutic option for drug-resistant infections though research on their optimal selection and clinical application remains in early stage (32, 33). This study aims to establish an optimized phage cocktail protocol against drug-resistant *Salmonella* and evaluate its clinical therapeutic efficacy using murine model. The isolated phage cocktail TSP_TW2, TSP_SW1, and TSP_SJ5 is capable of lysing common serotypes of *Salmonella* and *E. coli,* and demonstrates a significant synergistic antibacterial effect against *Salmonella* when used in combination with CIP (*P* < 0.001). This novel combination therapy not only effectively maintains intestinal microbiota balance and delays the emergence of phage resistance but also enhances therapeutic efficacy while reducing the minimum effective antibiotic dosage from 4 μg/mL to 1 μg/mL, providing an optimized strategy for the clinical management of drug-resistant *Salmonella* infections. Its cross-species broad-spectrum activity indicates potential for application in controlling a wider range of bacterial infections. *S*. Typhimurium poses significant public health threats as a hypervirulent serovar, characterized by multidrug resistance and fatal systemic infections. Epidemiological evidence identifies meat products as key transmission vehicles via farm-to-table processing chains (34). Antimicrobial resistance surveillance demonstrates broad cross-resistance to core antibiotics (β-lactams, fluoroquinolones, aminoglycosides), with genomic data revealing persistently increasing resistance gene prevalence (35, 36). Pathogenically, effector networks encoded by SPI-1/2 virulence islands precisely orchestrate host cell invasion and intracellular persistence, driving invasive sepsis (37, 38). Notably, this serovar exhibits significantly higher case fatality rates in HIV/AIDS patients and malnourished children compared to other *Salmonella* serovars (39), establishing it as a priority target for resistance surveillance and critical infection control.

The phylogenetic tree constructed based on the main capsid protein. These three phages belong to *Felixounavirus* genes (Fig. 1D). Genomic analysis revealed that although these phage genomes share similar sizes, they exhibit distinct lytic spectra. A systematic comparative analysis of the tail fiber proteins from the two highly homologous *Salmonella* phages TW1 and SW2 revealed significant differences in sequence length, domain composition, and the C-terminal receptor-binding region, which may result in distinct efficiencies or modes of interaction with host surface receptors. Studies have confirmed that these phenotypic differences likely stem from functional divergence of the tail fiber proteins, directly affecting their recognition and binding to host cells (40–42). The phages TSP_TW2, TSP_SW1, and TSP_SJ5, isolated from livestock slaughterhouses and farms, demonstrated targeted and efficient infectivity against all aforementioned serovars, exhibiting a broader host lytic spectrum compared to previous studies (43). Characterization of biological properties revealed maintained high activity under elevated temperatures and extreme pH conditions, coupled with shorter latency periods and enhanced adsorption rates (Fig. S2) (44). Most critically, the phage cocktail demonstrates significant penetrative disruption of established *Salmonella* biofilms revealing its potential to sustain antimicrobial efficacy in complex food-processing environments (e.g., slaughter equipment surfaces, refrigeration temperatures). Genomic comparisons similarly revealed significant polymorphism in the tail fiber protein-coding regions among these phages despite their shared taxonomic classification. For this phage genus, lipopolysaccharide has been identified as the primary receptor for *Felixounavirus* phages (45, 46). Comparative analysis of seven *Felixounavirus* phages revealed significant variations in lytic activity against *S*. Typhi and *S*. Paratyphi among geographically distinct isolates (47). Among three *Felixounavirus* phages isolated from the same dairy farm, only 67% overlap was observed in their lytic spectra against 21 *Salmonella* strains (48). This finding is consistent with our results demonstrating that *Felixounavirus* phages exhibit marked host diversity, which likely stems from geographical divergence or critical genomic modifications, particularly structural variations in baseplates and tail proteins involved in host recognition (49, 50). Recent studies indicate that the infection strategy of *Salmonella* phage Gifsy-1 is entirely dependent on the presence of the host O-antigen. In strains lacking the O-antigen, Gifsy-1 achieves multi-receptor adsorption through outer membrane proteins (such as OmpC) and the core oligosaccharide; whereas when the O-antigen is present, it switches to utilizing the core oligosaccharide as the sole receptor (51). This finding suggests that structural differences in the O-antigen can not only finely modulate the host range but may also fundamentally alter the phage’s receptor utilization mode. Therefore, the differences in the host ranges of TSP_TW2, TSP_SW1, and TSP_SJ5 may stem from variations in the recognition of O-antigen epitopes and/or involve the participation of alternative receptors such as outer membrane proteins.

Oromí-Bosch et al. (52) proposed that phage therapy for overcoming bacterial resistance evolution should incorporate the following strategies: (i) minimizing bacterial resistance, (ii) driving bacterial evolution toward favorable trade-offs, (iii) re-sensitizing bacteria to antibiotics, and (iv) steering evolution toward other reduced-virulence traits. Our study demonstrates successful implementation of strategies (i) and (iii) through the phage cocktail-antibiotic combination. This finding strongly corroborates the work of Oechslin et al (53) demonstrating that phage combination therapy can suppress resistant strain proliferation. *In vitro* killing curve assays demonstrated that phage cocktails delayed the emergence of bacterial resistance by up to 24 h under sustained selective pressure from diverse *Salmonella* serotypes compared to monophage treatments. Our analysis of the tail proteins from three phages (TSP_TW2, TSP_SW1, and TSP_SJ5) in the cocktail revealed significant amino acid sequence polymorphism within the distal tail proteins, substrate proteins, and particularly within the C-terminal receptor-binding domain of the primary receptor-binding protein—the tail filament protein. The amino acid polymorphism in the tail fiber proteins of these phages, by inducing structural differences in their receptor-binding domains, enables the multiphage components in the cocktail to exert a composite selective pressure targeting heterogeneous surface receptors. This pressure significantly raises the genetic barrier to the concurrent evolution of cross-resistance, thereby constituting the key mechanism for the synergistic effect and suppression of resistance. *Salmonella* dynamically counters phage stress through the synergistic action of multi-layered defense mechanisms. Among these, the opvAB-mediated phase variation of the O antigen serves as the core epigenetic regulation (54). It spontaneously generates receptor heterogeneity within the population, allowing susceptible and resistant subpopulations to coexist. Although this strategy does not completely block infection, it avoids the fitness cost associated with fixed resistance mutations, thereby safeguarding the population’s long-term adaptive potential. Once phages breach the adsorption barrier, intracellular defenses are initiated，the restriction-modification system cleaves non-methylated phage DNA, while abortive infection and toxin-antitoxin systems further protect the community by inducing infected cell death or a dormant/persister state, collectively forming a hierarchical defense network. Based on this, we preliminarily speculate that the advantage of the phage cocktail therapy observed in this study may stem from its multi-target strategy exploiting the intrinsic trade-offs among these defense mechanisms, indirectly delaying the emergence of complete resistance.

An emerging area in phage therapy research involves investigating whether phages can restore antibiotic sensitivity in drug-resistant bacteria. As representative example, combinatorial treatment employing phages with CIP/gentamicin exhibits significantly enhanced eradication efficacy against *Staphylococcus aureus* biofilms compared to monotherapeutic approaches (55), while phage cocktail-vancomycin combinations demonstrate reliable bactericidal activity against 153 clinically isolated MDR *Pseudomonas aeruginosa* strains (13). These findings fully confirm the synergistic advantages and broad applicability of PAS in clinical treatment. In our murine intestinal infection model, phage treatment led to restored susceptibility of originally resistant strains to β-lactams, quinolones, and tetracyclines as determined by antimicrobial susceptibility testing. This phenomenon may arise from phage infection-induced reductions in bacterial lipopolysaccharide synthesis or impairment of outer membrane integrity, thereby enhancing the penetration of cell wall biosynthesis-targeting antibiotics (e.g., β-lactams) (56). Another example is the synergistic interaction observed between CIPHP 3 and the cell wall inhibitor ceftazidime, which may be attributed to the cephalosporin’s ability to stimulate bacterial metabolism, enhance cellular proliferation, and consequently increase phage particle production (57, 58). Furthermore, adaptive mutations conferring phage resistance (e.g., LPS biosynthesis deficiencies) trigger evolutionary trade-offs that result in compensatory restoration of antibiotic susceptibility (59). CIP, as a DNA gyrase inhibitor, may theoretically cause antagonistic effects by blocking bacterial DNA replication. Based on this, this study adopted a sequential administration strategy, namely adding 1 µg/mL CIP 20 min after phage infection. Utilizing phage-targeted pathogen infection can disrupt bacterial cell structure and enhance antibiotic penetration. Meanwhile, the stress response induced by sublethal doses of CIP may promote phage replication, inhibit different tolerant subpopulations within the bacterial population, and reduce the risk of cross-resistance. These findings suggest that the specific type of interaction in each phage-antibiotic combination is largely determined by both the antibiotic’s primary target (e.g., cell wall, protein synthesis, or nucleic acid metabolism) and the host cellular processes essential for phage replication (e.g., DNA replication, transcription, or energy metabolism) (21, 60, 61). Collectively, these findings provide crucial experimental groundwork for further investigation into the molecular basis of PAS, while also highlighting potential avenues for developing more effective anti-resistance strategies.

The gut microbiota is recognized as a biomarker of intestinal health, with its most prominent function being the regulation of local and systemic immune system development and functional balance (10). This study revealed significant microbial dysbiosis in the antibiotic-treated group, compared to the phage cocktail and combination therapy groups, the antibiotic group exhibited statistically significant reductions in both Chao1 and Shannon indices (*P* < 0.001), concomitant with increased abundance of opportunistic pathogens (e.g., *Alloprevotella*, *Desulfovibrio*), and depletion of beneficial taxa (e.g., Muribaculaceae, *Lactobacillus*, *Bacteroides*). These results not only confirm the disruptive effects of antibiotics on microbial diversity but also demonstrate their profound restructuring of community architecture. Numerous studies have demonstrated that antibiotics exert substantial impacts on both the composition and functionality of the gut microbiota. For instance, vancomycin, a Gram-positive-specific antibiotic, paradoxically depletes Gram-negative bacterial populations, demonstrating that antibiotics exert broader impacts on indigenous gut microbiota than predicted by their antimicrobial spectra (62). Similarly, ampicillin-induced dysbiosis alters host-bacterial interactions and leads to changes in colonic sensation and motility in mice, accompanied by a reduction in *Bifidobacterium* spp. abundance (11). The differential impact of the phage-antibiotic combination on gut microbiota, compared to antibiotic monotherapy, may stem from its synergistic specificity. While CIP alone broadly suppresses commensals, the combination achieves rapid pathogen clearance at a lower effective antibiotic dose. This reduces the duration of broad-spectrum pressure and facilitates the reoccupation of the vacated niche by native beneficial bacteria, thereby promoting microbiota resilience. Of particular note, the combined phage cocktail-CIP therapeutic strategy in this study demonstrated unique microbiota protective advantages selectively eliminating pathogens (including resistant strains) while preserving commensal flora and significantly enhancing colonization of probiotic bacteria such as *Lactobacillus* and *Bacteroides*. These findings align with reports by Bao et al. using lysogenic phages to restore streptomycin induced dysbiosis (63, 64), collectively demonstrating the distinctive value of phage therapy in maintaining microbial homeostasis.

### Conclusion

This study successfully validated the significant therapeutic efficacy of phage cocktail therapy against drug-resistant *S*. Typhimurium infections. The selected phage cocktail exhibited broad-spectrum lytic activity and demonstrated synergistic antimicrobial effects when combined with CIP *in vivo*, effectively maintaining gut microbiota homeostasis while delaying resistance development. Importantly, this approach achieved simultaneous pathogen eradication and partial restoration of antibiotic susceptibility in resistant strains, demonstrating substantial clinical potential. These findings confirm the feasibility of phage therapy as either an alternative or adjunct to antibiotics, providing novel conceptual and experimental foundations for addressing the growing challenge of antimicrobial resistance.

## Data Availability

The phage whole genome data have been uploaded to the NCBI database (PV550667, PV550668, and PV550669), and the current date of the corresponding author’s setup is not yet publicly available. The corresponding data can be obtained from the first author or corresponding author upon request.

## SUPPLEMENTAL MATERIAL

Supplemental material spectrum.02640-25-s0001.docx Fig. S1 to S6; Tables S1 to S5.

ASM does not own the copyrights to Supplemental Material that may be linked to, or accessed through, an article. The authors have granted ASM a non-exclusive, world-wide license to publish the Supplemental Material files. Please contact the corresponding author directly for reuse.
