# Supplementary material for: Systematic evaluation of phage cocktail-ciprofloxacin combination therapy against multidrug-resistant Salmonella Typhimurium induced gut dysbiosis
Source: Microbiol Spectr. 2026 Mar 23;14(5):e02640-25. doi: 10.1128/spectrum.02640-25 (PMC13141883; doi:10.1128/spectrum.02640-25)
Supplement: Supplemental material — Fig. S1 to S6; Tables S1 to S5. [file spectrum.02640-25-s0001.docx]

Supplementary materials

**Systematic Evaluation of Phage Cocktail-Ciprofloxacin Combination Therapy** **Against** **Multidrug-Resistant** ***Salmonella*** **Typhimurium-Induced Gut Dysbiosis**

Qian Chong, Mingxia Chen, Qing Cao, Jiabin He, Zhonglong Wang, Jiayu Wang, Kunzhong Zhang, Huitian Gou^*^.

College of Veterinary Medicine, Gansu Agricultural University, LanZhou 730000, Gansu,China


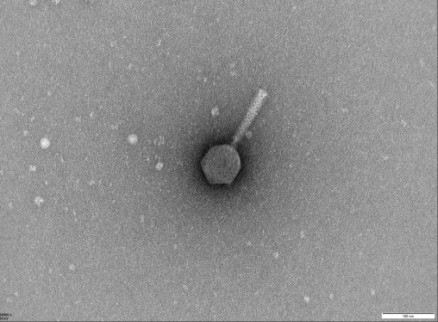


**TSP_SJ5**


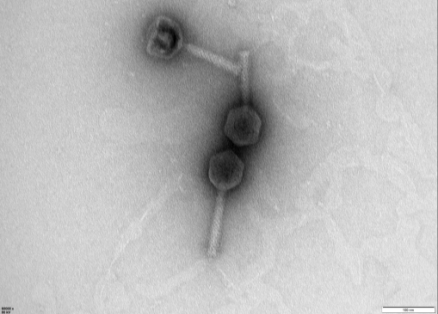


**TSP_SJ5**


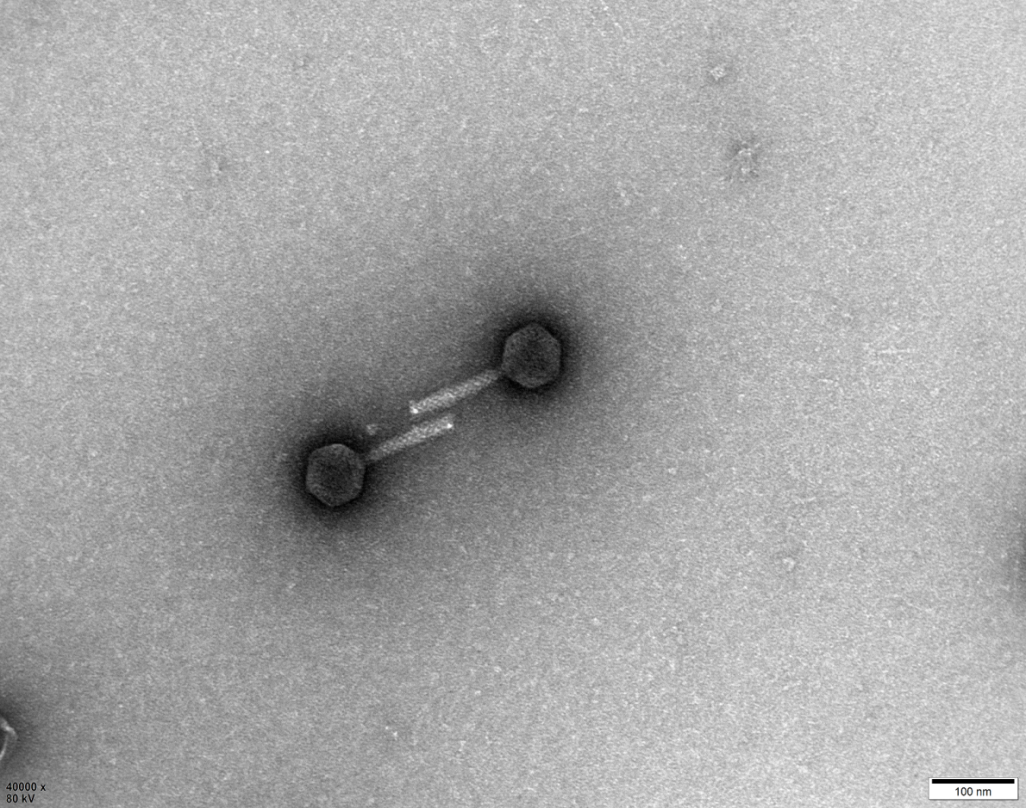


**TSP_SJ5**


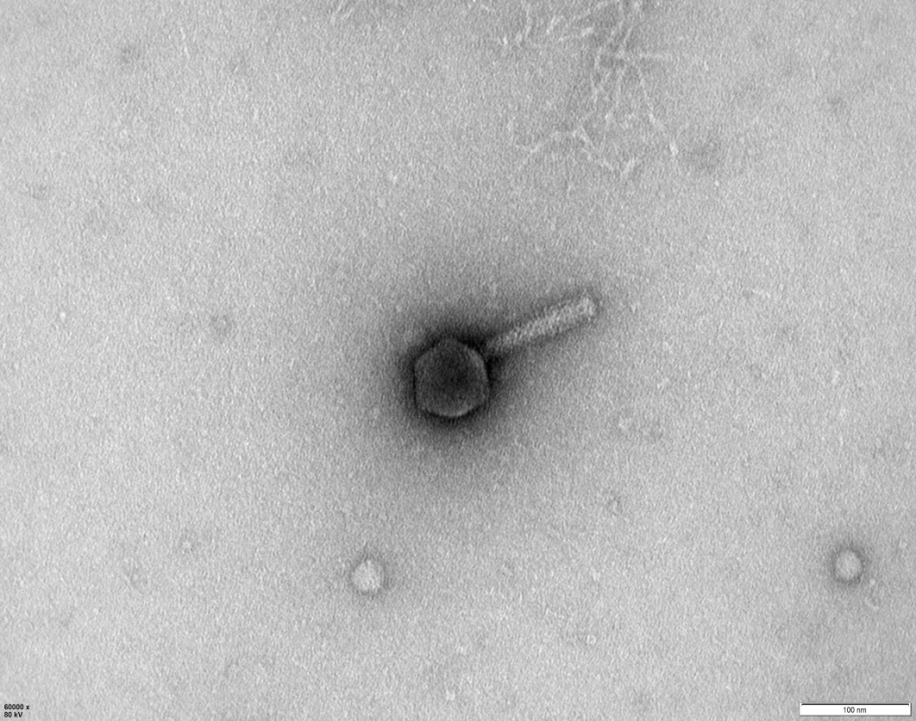


**TSP_SW1**


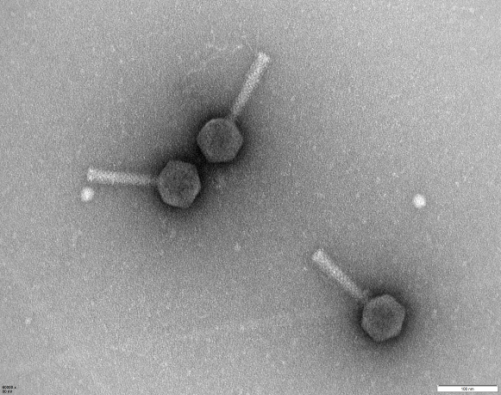


**TSP_TW2**


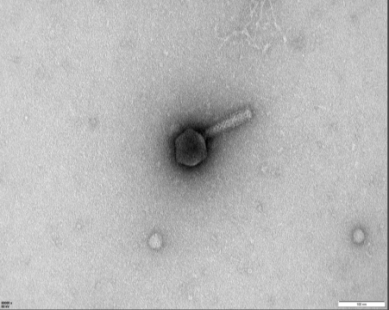

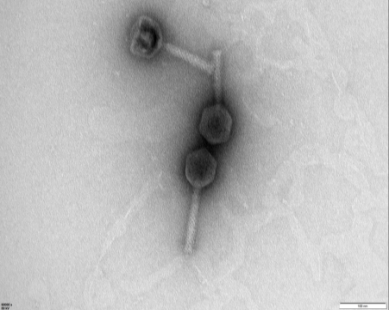

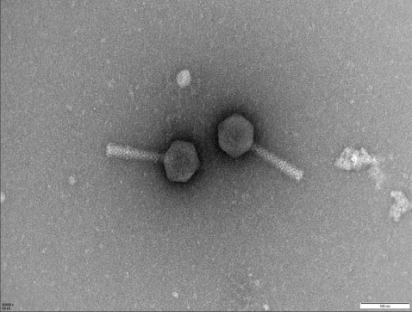

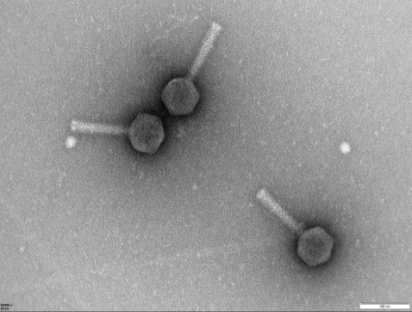


**TSP_SW1**

**TSP_SW1**

**TSP_TW2**

**TSP_TW2**

**Supplementary Fig S1. Transmission electron microscopy images of phages TSP_SJ5, TSP_SW1, and TSP_TW2.**


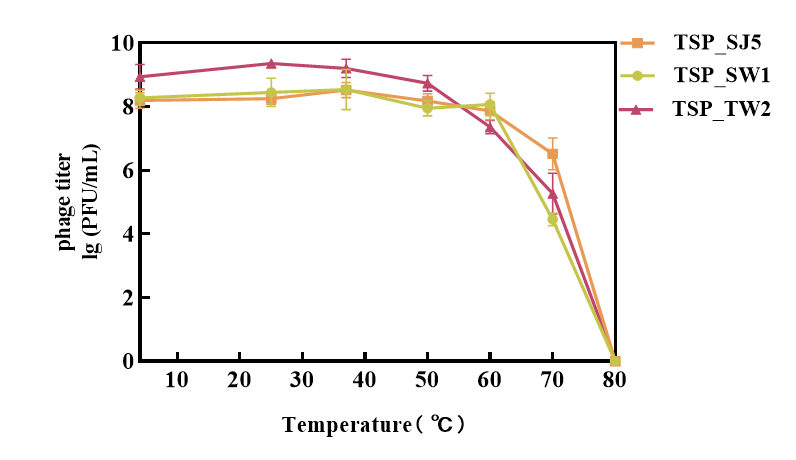

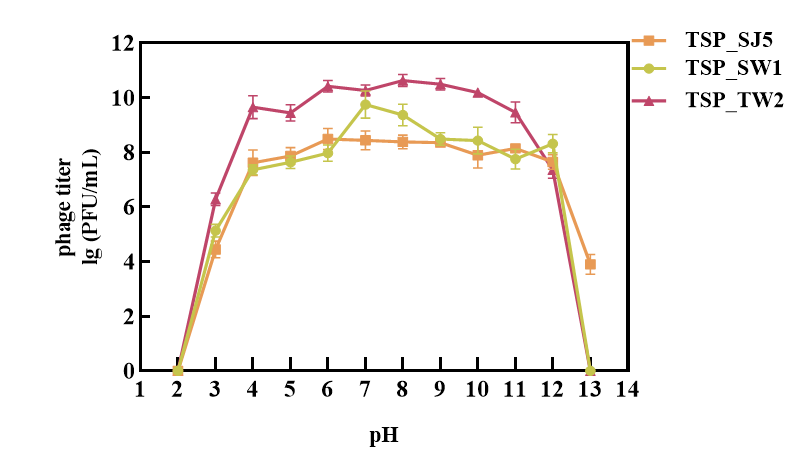

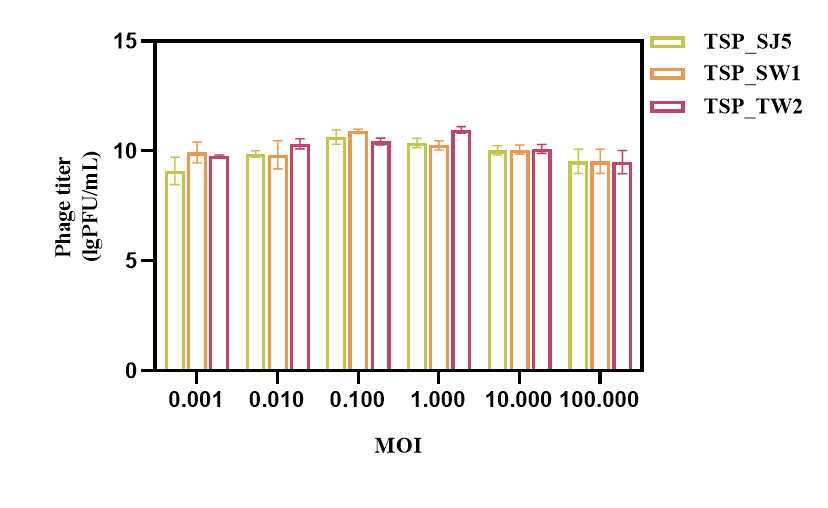


**A**

**B**

**C**

**D**

**Supplementary Fig S2. Characterization of biological properties of phages TSP_SJ5, TSP_SW1, and TSP_SW2. (A)** For thermal stability analysis, purified phage suspensions were aliquoted (1 mL/tube) and incubated at eight temperature gradients for 60 min, followed by infectivity assessment via double-layer agar assay at optimal MOI. **(B)** pH stability was evaluated by incubating phages in SM buffer (pH 1-14) at 37°C for 2 h prior to infectivity quantification at optimal MOI. **(C)** One-step growth curves were determined by infecting logarithmic-phase host bacteria (CVCC50115, OD_600_=0.6) at optimal MOI with 10-min sampling intervals during 37°C shaking incubation. **(D)** Optimal MOI was determined through serial dilution, where phage-host mixtures were centrifuged (10,000 rpm, 30 min, 4°C) after 10-min adsorption to remove unbound phages before titer quantification. All experiments were performed in triplicate with error bars representing SD. Data were analyzed by one-way ANOVA with Tukey's post hoc test (ns, not significant; **P* < 0.05; ***P* < 0.01; ****P* < 0.001).


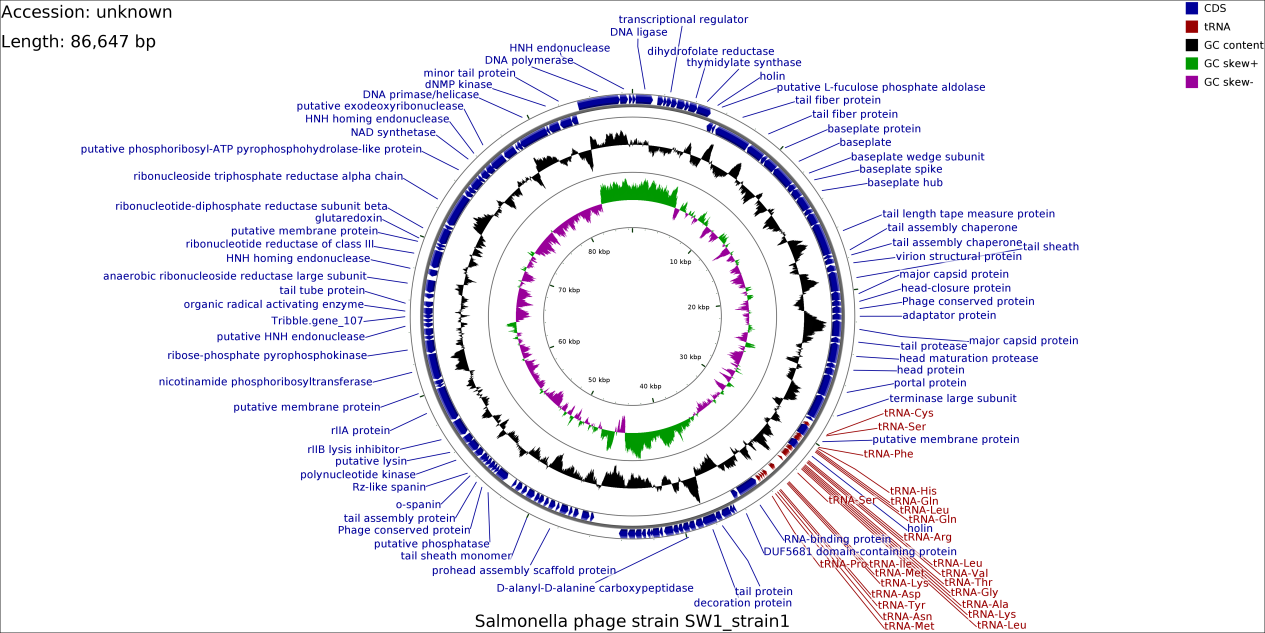

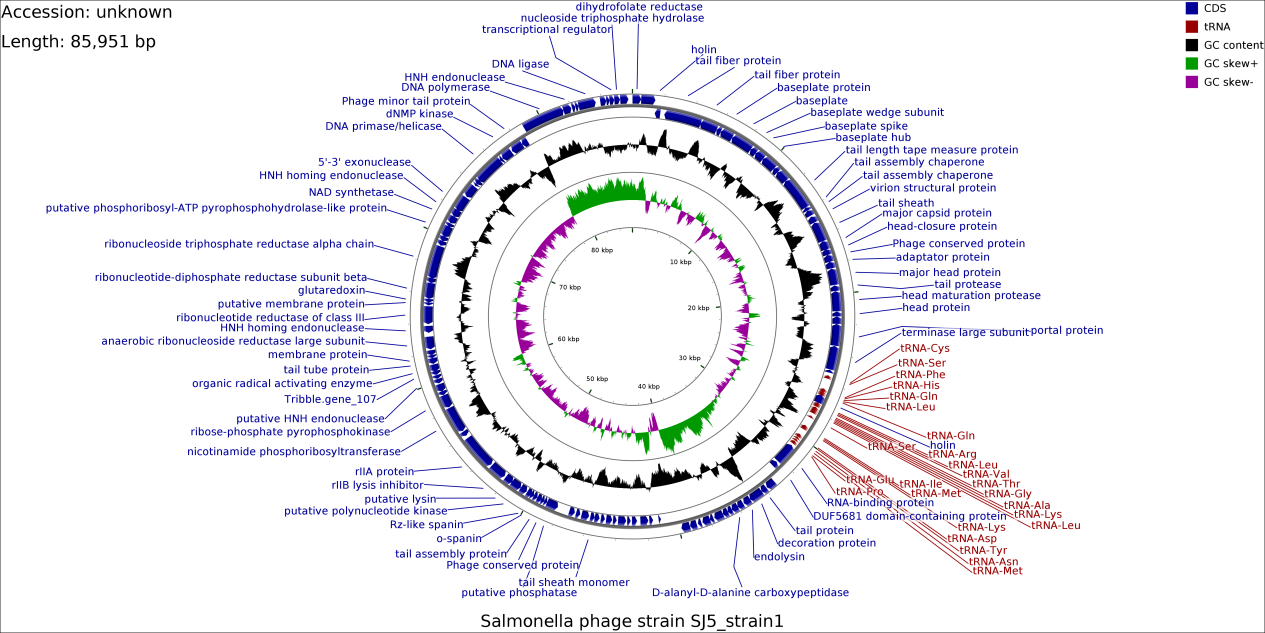

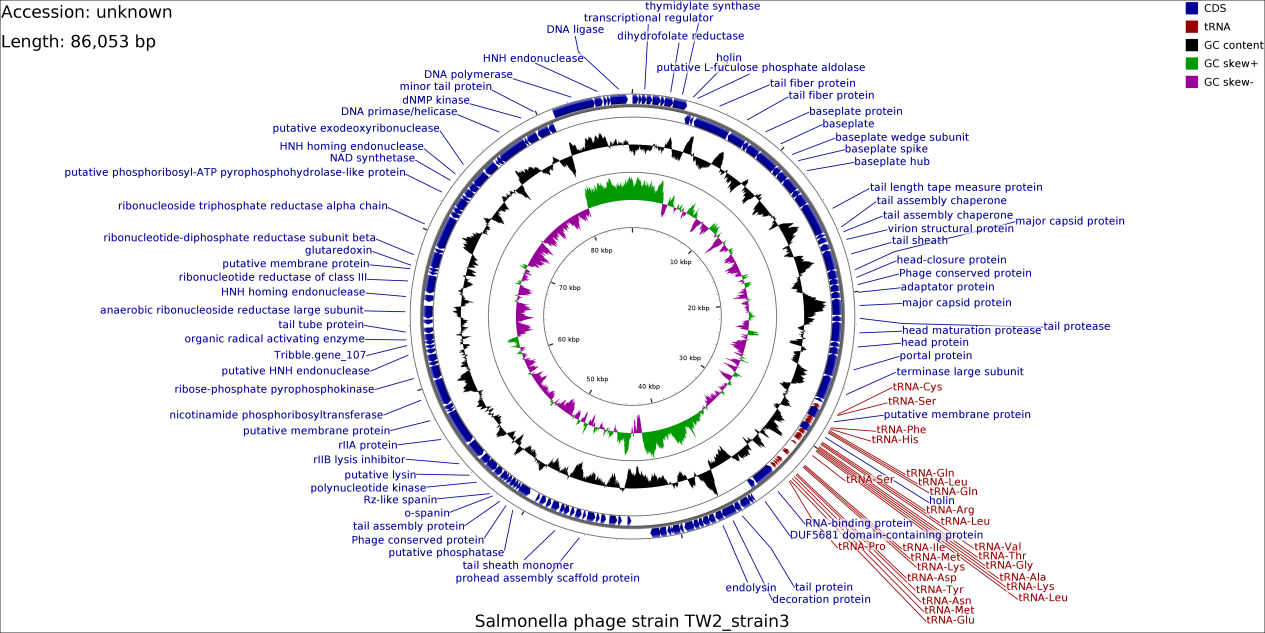


A

B

C

Supplementary **Fig S3. Genomic** circular **maps of the phages. (A)** Circular genome map of phage TSP_SW1 encoding 150 ORFs; **(B)** Circular genome map of phage TSP_TW2 encoding 145 ORFs; **(C)** Circular genome map of phage TSP_SJ5 encoding 148 ORFs.

C

B

A


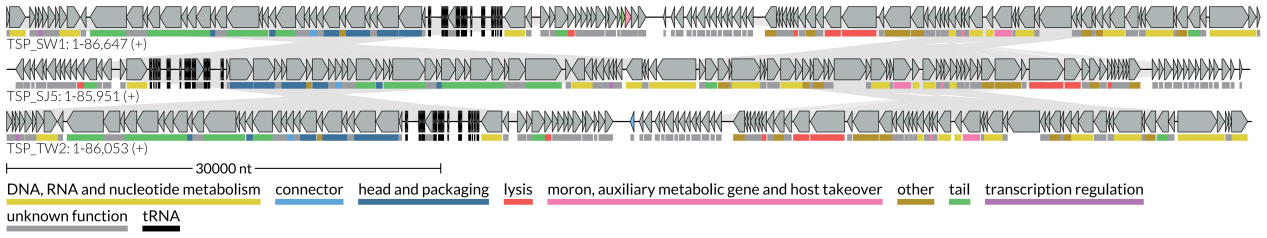


**Supplementary Fig. S4 Genomic Co-linearity Analysis of phages TSP_SW1, TSP_TW2, and TSP_SJ5.** Perform homology analysis of the complete genomes of three phages using Lovis4 software.

**A**

**B**

**Supplementary Fig S5. Effects of antimicrobial agents on phage titer and Salmonella MIC determination.** **(A)** Impact of ciprofloxacin on phage titer and Salmonella MIC testing; **(B)** Impact of tetracycline on phage titer and Salmonella MIC testing.


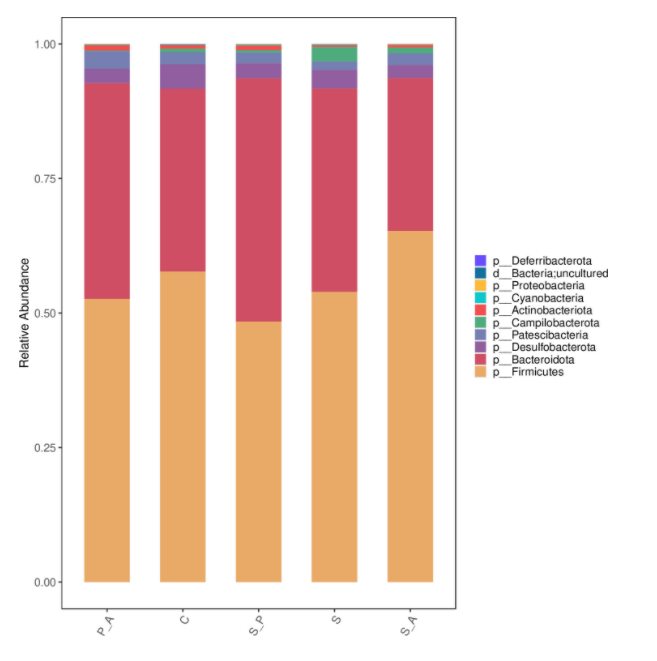


**Supplementary Fig S6 Bray-Curtis similarity clustering analysis of gut bacterial composition at the phylum level.** Phage-antibiotic cocktail (PA); PBS control (C); Phage cocktail treatment (SP); S. Typhimurium CMCC 50115 challenge group (S); Ciprofloxacin treatment (SA). Note: x-axis represents sample names, y-axis indicates relative abundance.

**Supplementary TableS1. The bacterial strains used in the experiment**

| Bacterial Serotype | Bacterial | Source |
| --- | --- | --- |
| S.Choleraesuis | SC_83 | Poultry |
| S.Choleraesuis | SC_D40-1 | Poultry |
| S.Pullorum | CMCC 1792 | Poultry |
| S.Pullorum | SP1 | Poultry |
| S.Typhi | CMCC 50115 | China Medical Culture Collection |
| S.Enteritidis | SE_SC6 | Poultry |
| S.Enteritidis | SE_SC7 | Poultry |
| S.Enteritidis | SE_SC12 | Poultry |
| S.Enteritidis | SE_SC9 | Poultry |
| S.Enteritidis | SE_SC20 | Poultry |
| S.Enteritidis | SE_SC27 | Poultry |
| S.Enteritidis | SE_SC174 | Poultry |
| S.Enteritidis | SE_SC178 | Poultry |
| S.Derby | SD_F3 | Dairy Farm |
| S.Derby | SD_F4 | Dairy Farm |
| S.Derby | SD_SD12 | Dairy Farm |
| S.Derby | SD_SD10 | Dairy Farm |
| S.Derby | SD_D32-1 | Dairy Farm |
| S.Derby | SD_20 | Dairy Farm |
| S.Derby | SD_114 | Sheep Farm |
| S.Derby | SD_21 | Sheep Farm |
| S.Derby | SD_17-2 | Sheep Farm |
| S.Derby | SD_57-1 | Dairy Farm |
| S.Derby | SD_50-2 | Dairy Farm |
| S.Rissen | SR_67-1 | Dairy Farm |
| S.Rissen | SR_266 | Dairy Farm |
| S.Rissen | SR_SD16 | Poultry |
| S.Rissen | SR_z24 | Poultry |
| S.Rissen | SR_D31 | Poultry |
| S.Rissen | SR_D21 | Poultry |
| S.Rissen | SR_D34-1 | Poultry |
| S.Rissen | SR_D27-2 | Poultry |
| S.Rissen | SR_35 | Poultry |
| S.Rissen | SR_31 | Poultry |
| S.Rissen | SR_36-1 | Poultry |
| S.Paratyphi C | SP_2-1 | Poultry |
| S.Paratyphi C | SP_35-1-1 | Poultry |
| S.Paratyphi C | SP_T12-2 | Poultry |
| S.Paratyphi C | SP_63-1 | Poultry |
| S.Paratyphi C | SP_38 | Poultry |
| S.Paratyphi C | SP_36 | Poultry |
| Bacterial Serotype | Bacterial | Source |
| S.Paratyphi C | SP_17-2 | Poultry |
| S.Paratyphi C | SP_K49-1 | Poultry |
| S.Paratyphi C | SP_D40-1 | Dairy Farm |
| S.Typhimurium | ST_z68 | Dairy Farm |
| S.Typhimurium | ST_6 | Dairy Farm |
| S.Typhimurium | ST_38-1 | Dairy Farm |
| S.Typhimurium | ST_68 | Dairy Farm |
| S.Typhimurium | ST_T7 | Dairy Farm |
| S.Typhimurium | ST_T4 | Dairy Farm |
| S.Typhimurium | ST_T3 | Dairy Farm |
| S.Typhimurium | ST_T6 | Dairy Farm |
| S.Typhimurium | ST_z11 | Dairy Farm |
| S.Typhimurium | ST_N13-1 | Dairy Farm |
| S.Typhimurium | ST_N19-2 | Dairy Farm |
| S.Typhimurium | ST_N14-1 | Dairy Farm |
| S.Typhimurium | ST_N18-2 | Dairy Farm |
| S.Typhimurium | ST_N18-1 | Dairy Farm |
| S.Typhimurium | ST_N35-1 | Dairy Farm |
| S.Typhimurium | ST_N15-2 | Dairy Farm |
| S.Typhimurium | ST_N34-2 | Dairy Farm |
| S.Typhimurium | ST_N37-1 | Dairy Farm |
| S.Typhimurium | ST_N19-1 | Dairy Farm |
| S.Typhimurium | ST_N34-1 | Dairy Farm |
| S.Typhimurium | ST_D01-2 | Poultry |
| S.Typhimurium | ST_D44 | Poultry |
| S.Typhimurium | ST_12-2 | Poultry |
| S.Typhimurium | ST_K49-2 | Poultry |
| S.Typhimurium | ST_3-1 | Poultry |
| S.Typhimurium | ST_3-2 | Poultry |
| S.Typhimurium | ST_TW06-1 | Poultry |
| S.Thompson | STh_D15 | Poultry |
| S.Thompson | STh_D7 | Poultry |
| E.coli | BL21 | Laboratory stock |
| L.monocytogenes | ATCC 19115 | American Type Culture Collection |
| L.monocytogenes | ATCC 19112 | American Type Culture Collection |
| L.monocytogenes | ATCC 19111 | American Type Culture Collection |
| L.monocytogenes | ATCC 19890 | American Type Culture Collection |

**Supplementary TableS2. Sources of 24 phage isolates**

| Phage | Source |
| --- | --- |
| SSP_SJ5 | Poultry Sewage |
| TSP_SW4 | Poultry Sewage |
| SSP_TW2 | Poultry Sewage |
| ESP_W3 | Poultry Sewage |
| TSP_W7 | Poultry Sewage |
| SSP_YN1 | Poultry Sewage |
| ESP_YN2 | Poultry Sewage |
| CSP_YN5 | Poultry Sewage |
| TSP_YN6 | Poultry Sewage |
| ESP_YJ1 | Pigs Farm Sewage |
| SSP_YN9 | Dairy Farm Sewage |
| CSP_SJ8 | Dairy Farm Sewage |
| TSP_SJ5 | Dairy Farm Sewage |
| TSP_SJ1 | Dairy Farm Sewage |
| TSP_SJ9 | Dairy Farm Sewage |
| TSP_YJ1 | Pigs Farm Sewage |
| TSP_YJ2 | Pigs Farm Sewage |
| TSP_YJ4 | Pigs Farm Sewage |
| TSP_SW1 | Sheep Farm Sewage |
| ESP_SW2 | Sheep Farm Sewage |
| ESP_SW3 | Sheep Farm Sewage |
| ESP_SY2 | Poultry Sewage |
| ESP_SY3 | Poultry Sewage |
| ESP_SY5 | Poultry Sewage |

**Supplementary Table S3. Genome annotation of TSP_TW2 encoded proteins.**

|  | Functional protein |
| --- | --- |
| ORF 3 | transcriptional regulator |
| ORF 7 | dihydrofolate reductase |
| ORF 8 | thymidylate synthase |
| ORF 9 | holin |
| ORF 10 | putative L-fuculose phosphate aldolase |
| ORF 11 | tail fiber protein |
| ORF 12 | tail fiber protein |
| ORF 14 | baseplate protein |
| ORF 15 | baseplate |
| ORF 16 | baseplate wedge subunit |
| ORF 17 | baseplate spike |
| ORF 18 | baseplate hub |
| ORF 21 | tail length tape measure protein |
| ORF 22 | tail assembly chaperone |
| ORF 23 | tail assembly chaperone |
| ORF 24 | virion structural protein |
| ORF 25 | tail sheath |
| ORF 26 | major capsid protein |
| ORF 27 | head-closure protein |
| ORF 28 | Phage conserved protein |
| ORF 29 | adaptator protein |
| ORF 30 | major capsid protein |
| ORF 31 | tail protease |
| ORF 32 | head maturation protease |
| ORF 33 | head protein |
| ORF 35 | portal protein |
| ORF 36 | terminase large subunit |
| ORF 37 | unknown |
| ORF 40 | putative membrane protein |
| ORF 46 | holin |
| ORF 65 | RNA-binding protein |
| ORF 66 | DUF5681 domain-containing protein |
| ORF 70 | tail protein |
| ORF 71 | decoration protein |
| ORF 72 | endolysin |
| ORF 74 | D-alanyl-D-alanine carboxypeptidase |
| ORF 90 | prohead assembly scaffold protein |
| ORF 94 | tail sheath monomer |
| ORF 99 | putative phosphatase |
| ORF 100 | Phage conserved protein |
| ORF 102 | tail assembly protein |
| ORF 104 | o-spanin |
|  | Functional protein |
| ORF 105 | Rz-like spanin |
| ORF 107 | polynucleotide kinase |
| ORF 108 | putative lysin |
| ORF 109 | *rIIB* lysis inhibitor |
| ORF 110 | *rIIA* protein |
| ORF 111 | putative membrane protein |
| ORF 113 | nicotinamide phosphoribosyltransferase |
| ORF 114 | ribose-phosphate pyrophosphokinase |
| ORF 117 | putative HNH endonuclease |
| ORF 119 | Tribble.gene_107 |
| ORF 120 | organic radical activating enzyme |
| ORF 121 | tail tube protein |
| ORF 123 | anaerobic ribonucleoside reductase large subunit |
| ORF 124 | HNH homing endonuclease |
| ORF 125 | ribonucleotide reductase of class III |
| ORF 126 | putative membrane protein |
| ORF 127 | glutaredoxin |
| ORF 128 | ribonucleotide-diphosphate reductase subunit beta |
| ORF 130 | ribonucleoside triphosphate reductase alpha chain |
| ORF 131 | NAD synthetase |
| ORF 133 | HNH homing endonuclease |
| ORF 135 | putative exodeoxyribonuclease |
| ORF 136 | DNA primase/helicase |
| ORF 138 | dNMP kinase |
| ORF 139 | minor tail protein |
| ORF 141 | DNA polymerase |
| ORF 143 | HNH endonuclease |
| ORF 145 | DNA ligase |

**Supplementary Table S4. Genome annotation of TSP_SW1 encoded proteins.**

|  | Functional protein |
| --- | --- |
| ORF 2 | DNA ligase |
| ORF 5 | transcriptional regulator |
| ORF 9 | dihydrofolate reductase |
| ORF 10 | thymidylate synthase |
| ORF 11 | Holing |
| ORF 12 | putative L-fuculose phosphate aldolase |
| ORF 13 | tail fiber protein |
| ORF 14 | tail fiber protein |
| ORF 16 | baseplate protein |
| ORF 17 | Baseplate |
| ORF 18 | baseplate wedge subunit |
| ORF 19 | baseplate spike |
| ORF 20 | baseplate hub |
| ORF 23 | tail length tape measure protein |
| ORF 24 | tail assembly chaperone |
| ORF 25 | tail assembly chaperone |
| ORF 26 | virion structural protein |
| ORF 27 | tail sheath |
| ORF 28 | major capsid protein |
| ORF 29 | head-closure protein |
| ORF 30 | Phage conserved protein |
| ORF 31 | adaptator protein |
| ORF 32 | major capsid protein |
| ORF 33 | tail protease |
| ORF 34 | head maturation protease |
| ORF 35 | head protein |
| ORF 37 | portal protein |
| ORF 38 | terminase large subunit |
| ORF 42 | putative membrane protein |
| ORF 48 | holin |
| ORF 67 | RNA-binding protein |
| ORF 68 | DUF5681 domain-containing protein |
| ORF 72 | tail protein |
| ORF 73 | decoration protein |
| ORF 74 | endolysin |
| ORF 76 | D-alanyl-D-alanine carboxypeptidase |
| ORF 93 | prohead assembly scaffold protein |
| ORF 97 | tail sheath monomer |
| ORF 102 | putative phosphatase |
| ORF 103 | Phage conserved protein |
| ORF 105 | tail assembly protein |
| ORF 107 | o-spanin |
|  | Functional protein |
| ORF 108 | Rz-like spanin |
| ORF 110 | polynucleotide kinase |
| ORF 111 | putative lysin |
| ORF 112 | *rIIB* lysis inhibitor |
| ORF 113 | protein |
| ORF 114 | putative membrane protein |
| ORF 116 | nicotinamide phosphoribosyltransferase |
| ORF 117 | ribose-phosphate pyrophosphokinase |
| ORF 120 | putative HNH endonuclease |
| ORF 122 | Tribble.gene_107 |
| ORF 123 | organic radical activating enzyme |
| ORF 124 | tail tube protein |
| ORF 126 | anaerobic ribonucleoside reductase large subunit |
| ORF 127 | HNH homing endonuclease |
| ORF 128 | ribonucleotide reductase of class III |
| ORF 129 | putative membrane protein |
| ORF 130 | glutaredoxin |
| ORF 131 | ribonucleotide-diphosphate reductase subunit beta |
| ORF 133 | ribonucleoside triphosphate reductase alpha chain |
| ORF 136 | putative phosphoribosyl-ATP pyrophosphohydrolase-like protein |
| ORF 138 | NAD synthetase |
| ORF 139 | HNH homing endonuclease |
| ORF 140 | putative exodeoxyribonuclease |
| ORF 144 | DNA primase/helicase |
| ORF 146 | dNMP kinase |
| ORF 147 | minor tail protein |
| ORF 149 | DNA polymerase |
| ORF 150 | HNH endonuclease |

**Supplementary TableS5. Genome annotation of TSP_SJ5 encoded proteins.**

|  | Functional protein |
| --- | --- |
| ORF 1 | tail fiber protein |
| ORF 2 | tail fiber protein |
| ORF 3 | putative L-fuculose phosphate aldolase |
| ORF 4 | holin |
| ORF 5 | thymidylate synthase |
| ORF 6 | dihydrofolate reductase |
| ORF 10 | transcriptional regulator |
| ORF 13 | DNA ligase |
| ORF 16 | HNH endonuclease |
| ORF 17 | DNA polymerase |
| ORF 19 | minor tail protein |
| ORF 20 | dNMP kinase |
| ORF 22 | DNA primase/helicase |
| ORF 26 | putative exodeoxyribonuclease |
| ORF 27 | HNH homing endonuclease |
| ORF 28 | NAD synthetase |
| ORF 30 | putative phosphoribosyl-ATP pyrophosphohydrolase-like protein |
| ORF 33 | ribonucleoside triphosphate reductase alpha chain |
| ORF 35 | ribonucleotide-diphosphate reductase subunit beta |
| ORF 36 | glutaredoxin |
| ORF 37 | putative membrane protein |
| ORF 38 | ribonucleotide reductase of class III |
| ORF 39 | HNH homing endonuclease |
| ORF 40 | anaerobic ribonucleoside reductase large subunit |
| ORF 42 | tail tube protein |
| ORF 43 | organic radical activating enzyme |
| ORF 44 | Tribble.gene_107 |
| ORF 46 | putative HNH endonuclease |
| ORF 49 | ribose-phosphate pyrophosphokinase |
| ORF 50 | nicotinamide phosphoribosyltransferase |
| ORF 52 | putative membrane protein |
| ORF 53 | rIIA protein |
| ORF 54 | rIIB lysis inhibitor |
| ORF 55 | putative lysin |
| ORF 56 | polynucleotide kinase |
| ORF 58 | Rz-like spanin |
| ORF 59 | o-spanin |
| ORF 60 | hypothetical protein |
| ORF 61 | tail assembly protein |
| ORF 62 | Phage conserved protein |
| ORF 63 | putative phosphatase |
| ORF 67 | tail sheath monom |
|  | Functional protein |
| ORF 71 | prohead assembly scaffold protein |
| ORF 88 | D-alanyl-D-alanine carboxypeptidase |
| ORF 90 | endolysin |
| ORF 91 | decoration protein |
| ORF 92 | tail protein |
| ORF 96 | DUF5681 domain-containing protein |
| ORF 97 | RNA-binding protein |
| ORF 116 | holin |
| ORF 122 | putative membrane protein |
| ORF 126 | terminase large subunit |
| ORF 127 | portal protein |
| ORF 129 | head protein |
| ORF 130 | head maturation protease |
| ORF 131 | tail protease |
| ORF 132 | major capsid protein |
| ORF 133 | adaptator protein |
| ORF 134 | Phage conserved protein |
| ORF 135 | head-closure protein |
| ORF 136 | major capsid protein |
| ORF 137 | tail sheath |
| ORF 138 | virion structural protein |
| ORF 139 | tail assembly chaperone |
| ORF 140 | tail assembly chaperone |
| ORF 141 | tail length tape measure protein |
| ORF 144 | baseplate hub |
| ORF 145 | baseplate spike |
| ORF 146 | baseplate wedge subunit |
| ORF 147 | baseplate |
| ORF 148 | baseplate protein |
